# Supplementary material for: Supramolecular tessellations by the exo-wall interactions of pagoda[4]arene
Source: Nat Commun. 2021 Nov 4;12:6378. doi: 10.1038/s41467-021-26729-3 (PMC8568916; doi:10.1038/s41467-021-26729-3)
Supplement: Supplementary file 1 — Supplementary Information [file 41467_2021_26729_MOESM1_ESM.pdf]

Supplementary Information

Supramolecular Tessellations by the Exo-Wall Interactions of Pagoda[4]arene

Xiao-Ni Han,[a,b] Ying Han,[a] and Chuan-Feng Chen[a,b,*]

[a]*Beijing National Laboratory for Molecular Sciences, CAS Key Laboratory of Molecular Recognition and Function, Institute of Chemistry, Chinese Academy of Sciences, Beijing 100190, China.* [b]*University of Chinese Academy of Sciences, Beijing 100049, China.*

Email: cchen@iccas.ac.cn

Contents

1. **Synthesis and Characterization of P4 and Guests 2**
2. **NMR Spectra and NMR Titration Experiments 6**
3. **UV-vis Absorption Spectra of P4 with TPN and TFTN 15**
4. **Crystal Engineering of Pagoda[4]arene Crystals 16**
5. **Crystal Engineering of Pagoda[4]arene-Based Co-crystals 19**

1. Synthesis and Characterization of P4 and Guests

P4 was prepared according to reported literature (*J. Am. Chem. Soc.* 2020, **142**, 8262), and terephthalonitrile (TPN), tetrafluoroterephthalonitrile (TFTN) and 1,4-dinitrobenzene (DNB) were purchased and were used without further purification.

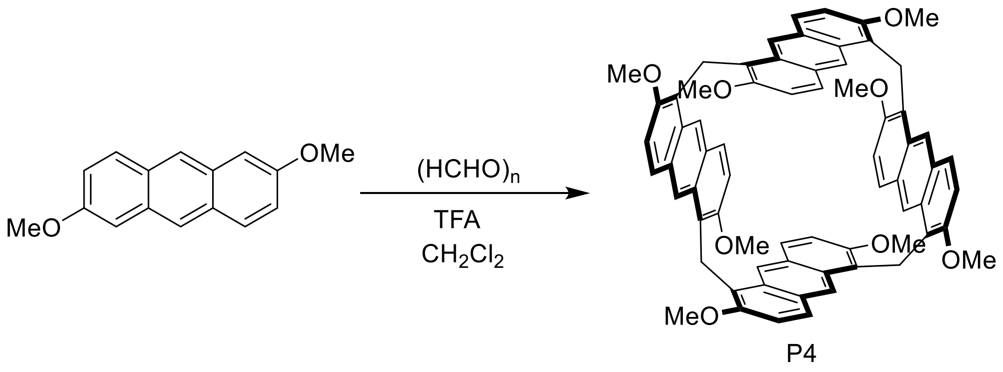

To a mixture of 2,6-dimethoxyanthracene (600 mg, 2.5 mmol) and paraformaldehyde (235 mg, 7.5 mmol) in dichloromethane (200 mL) was added TFA (95 μL, 1.25 mmol) under argon. The mixture was stirred at room temperature for 9 h. Then the reaction was quenched by the addition of 100 mL water. The organic layer was separated and dried with anhydrous $MgSO_4$. The solvent was removed in vacuo and the residue was separated by column chromatography on silica gel (eluent (TLC): 1.5/1 petroleum ether/EA, Rf = 0.4) to give P4 (194 mg, 31%) as yellow solid. mp: >280 °C. ^{1}H NMR (500 MHz, $CDCl_3$): δ 8.13 (s, 8H, Ar-H), 7.38 (d, J = 9.3 Hz, 8H, Ar-H), 7.19 (d, J = 9.3 Hz, 8H, Ar-H), 4.93 (s, 8H, CH_2), 4.20 (s, 24H, OMe). ^{13}C NMR (126 MHz, $CDCl_3$): δ 152.1, 129.9, 129.1, 128.5, 123.3, 122.0, 114.2, 57.1, 20.8. HRMS (APCI): *m/z* calcd for $[M+H]^+$ $C_{68}H_{57}O_8^+$: 1001.40480; found: 1001.40485.

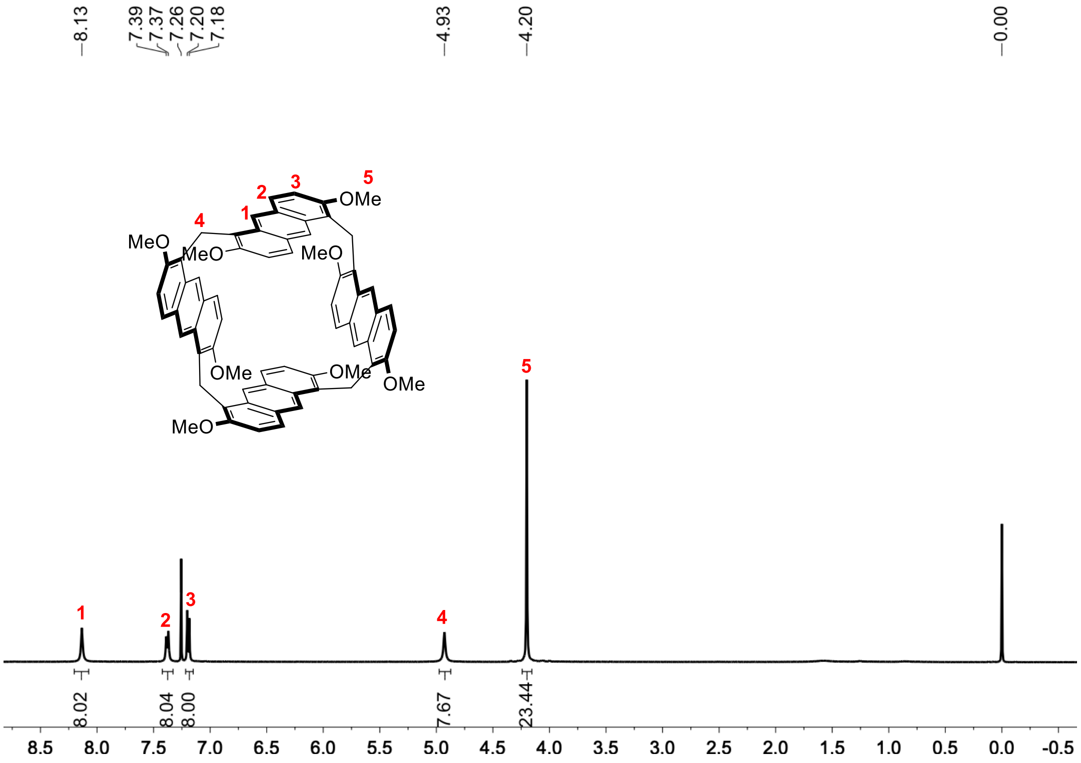

Supplementary Figure 1. ^{1}H NMR spectrum (500 MHz, $CDCl_3$, 298K) of P4.

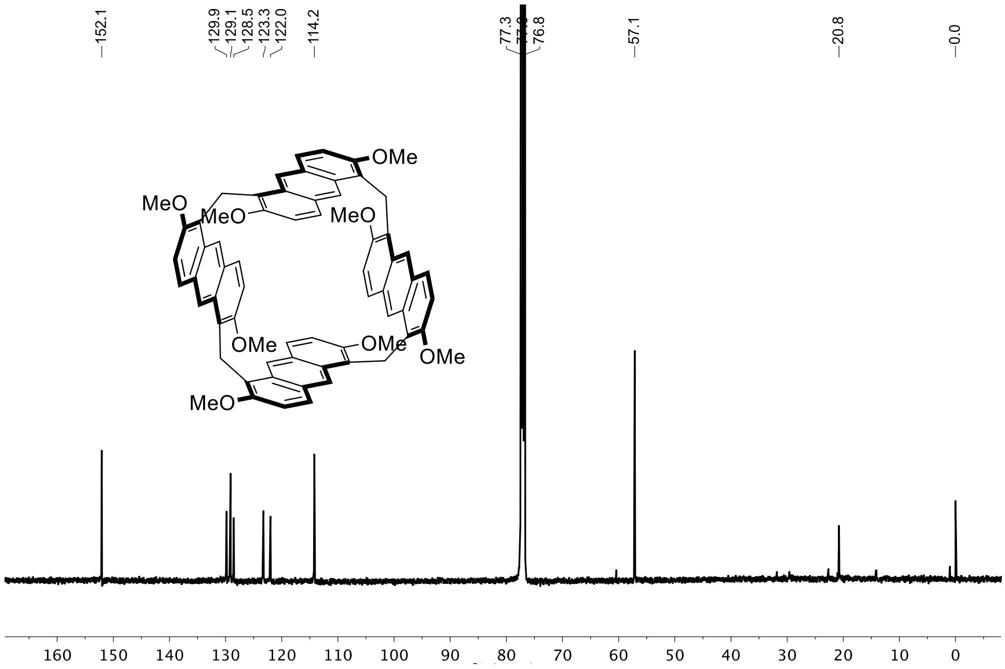

Supplementary Figure 2. ^{13}C NMR spectrum (126 MHz, $CDCl_3$, 298K) of P4.

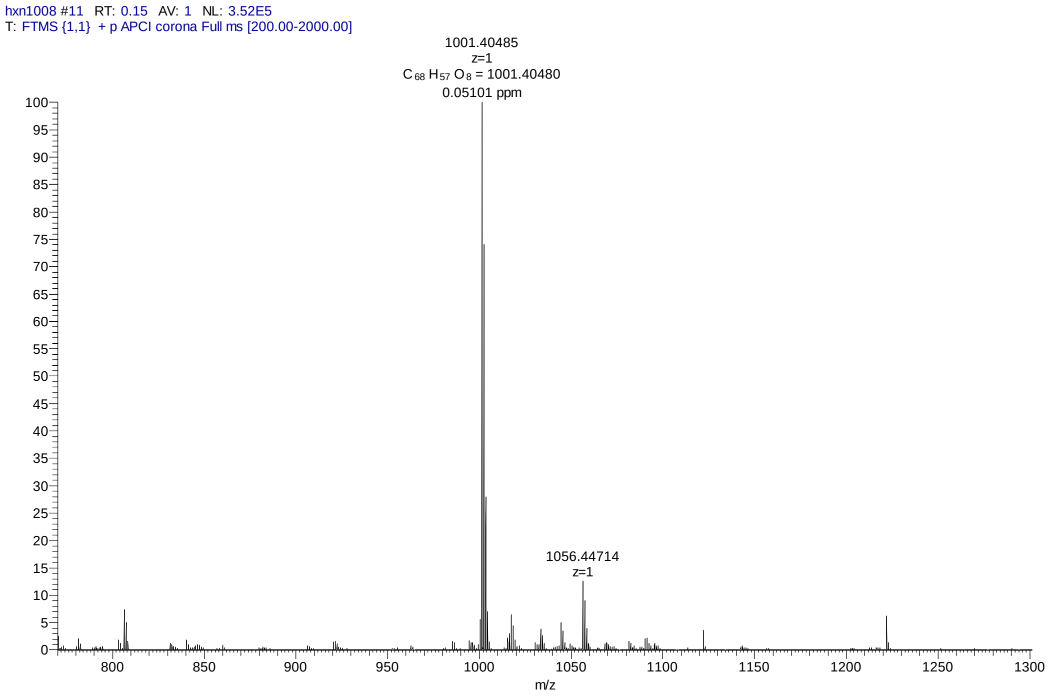

Supplementary Figure 3. HRMS spectrum (APCI) of P4.

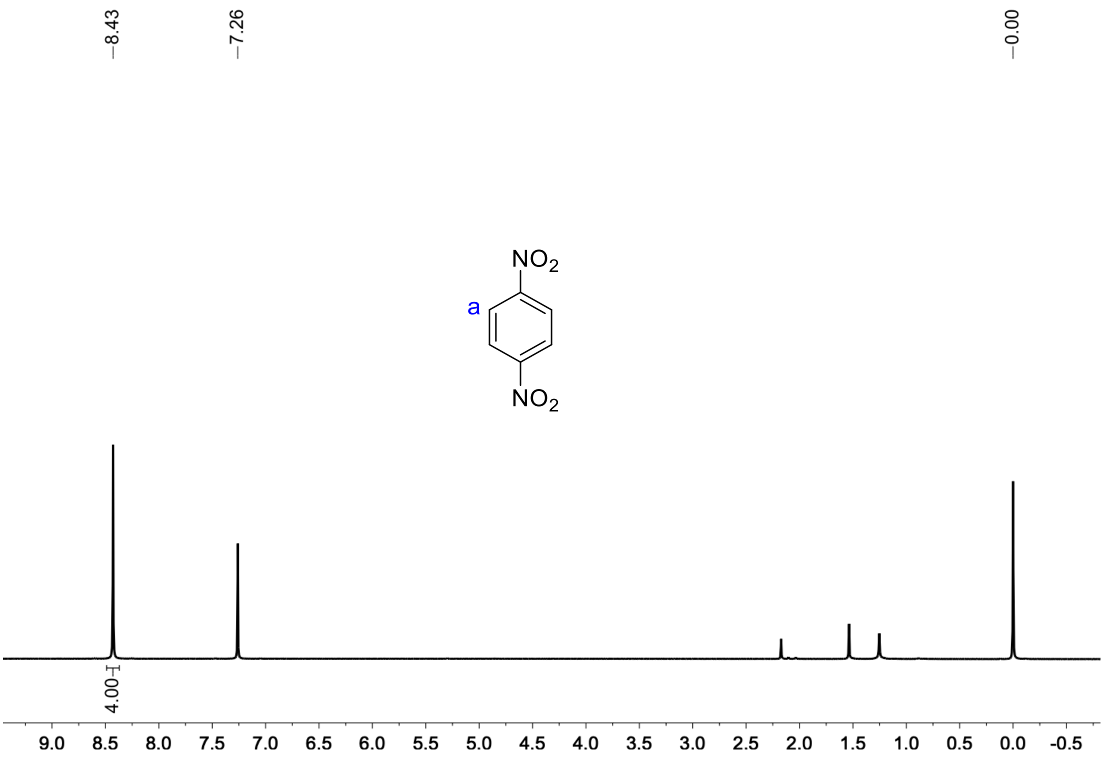

Supplementary Figure 4. ^{1}H NMR spectrum (500 MHz, $CDCl_3$, 298K) of DNB.

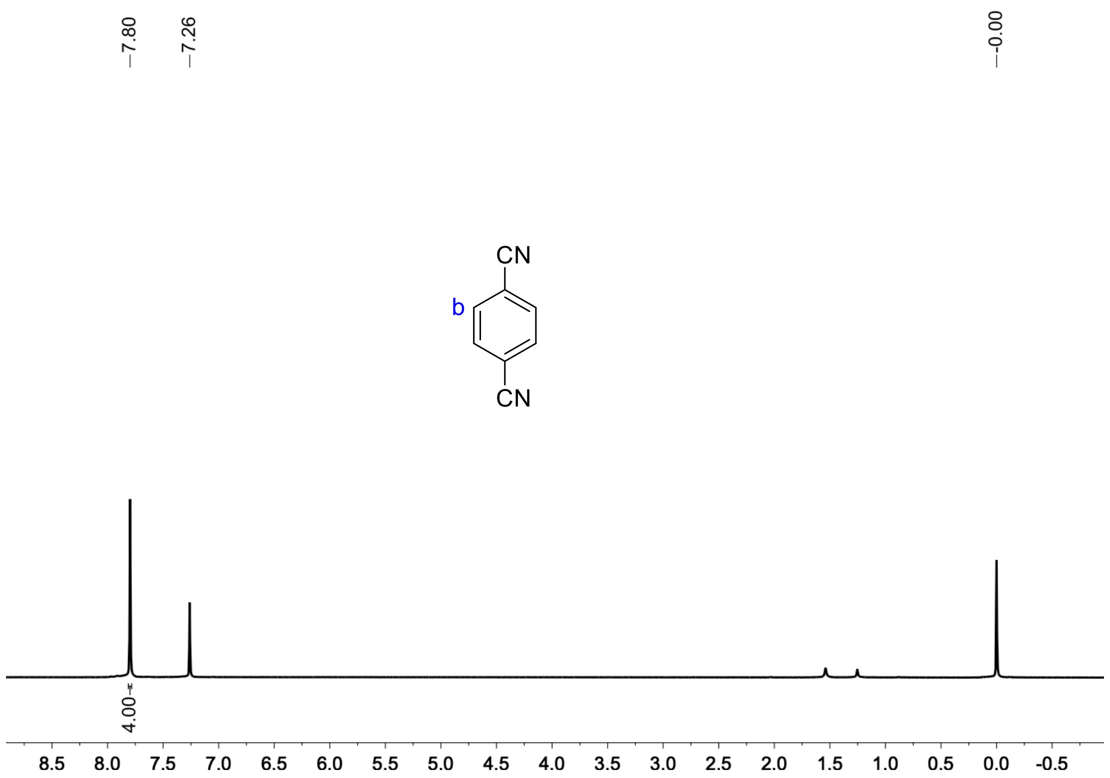

Supplementary Figure 5. ^{1}H NMR spectrum (500 MHz, $CDCl_3$, 298K) of TPN.

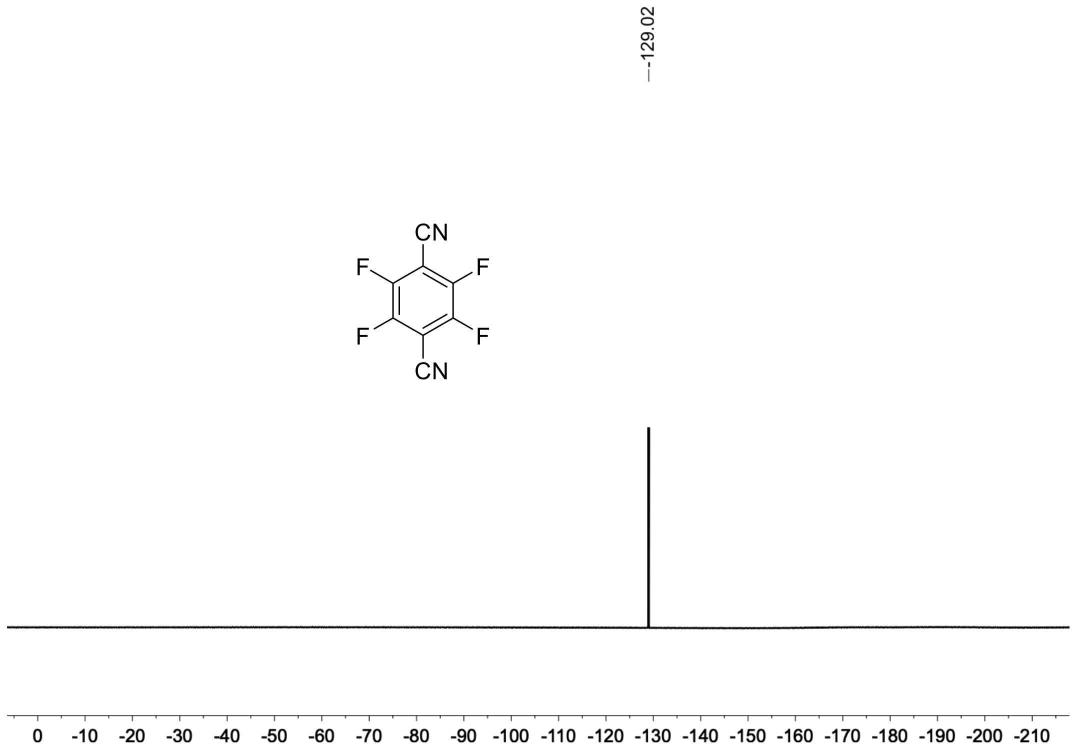

Supplementary Figure 6. ^{19}F NMR spectrum (377 MHz, CD_2Cl_2, 298K) of TFTN.

2. NMR Spectra and NMR Titration Experiments

2.1 ^1H and ^{19}F NMR Spectra

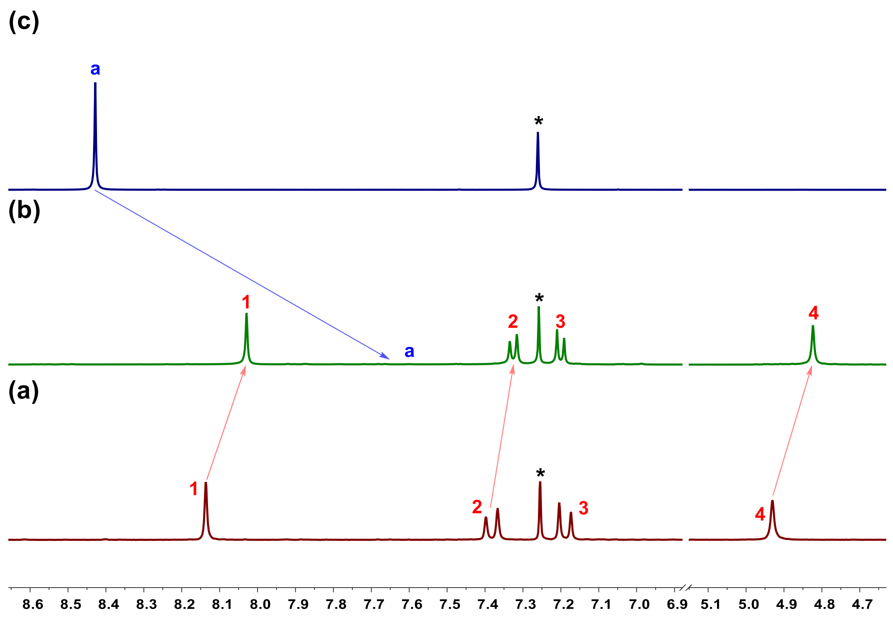

Supplementary Figure 7. Partial ^1H NMR spectra (500 MHz, $CDCl_3$, 298K) of (a) free P4 (5.0 mM), (b) P4 (5.0 mM) and DNB (10.0 mM), and (c) free DNB (5.0 mM).

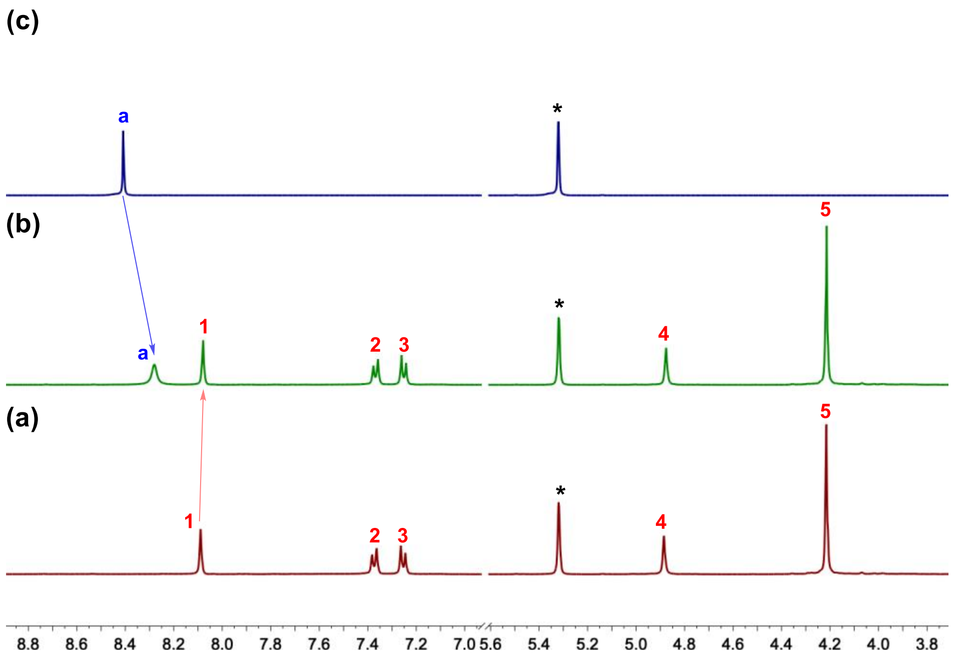

Supplementary Figure 8. Partial ^1H NMR spectra (500 MHz, CD_2Cl_2, 298K) of (a) free P4 (5.0 mM), (b) P4 (5.0 mM) and DNB (10.0 mM), and (c) free DNB (5.0 mM).

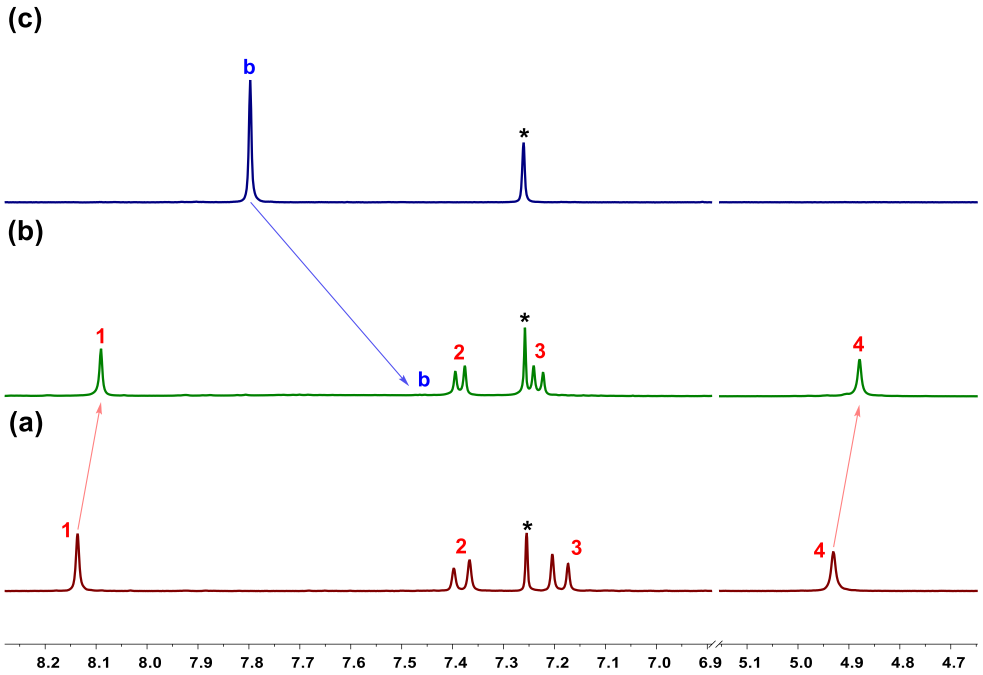

Supplementary Figure 9. Partial ^{1}H NMR spectra (500 MHz, $CDCl_3$, 298K) of (a) free P4 (5.0 mM), (b) P4 (5.0 mM) and TPN (10.0 mM), and (c) free TPN (5.0 mM).

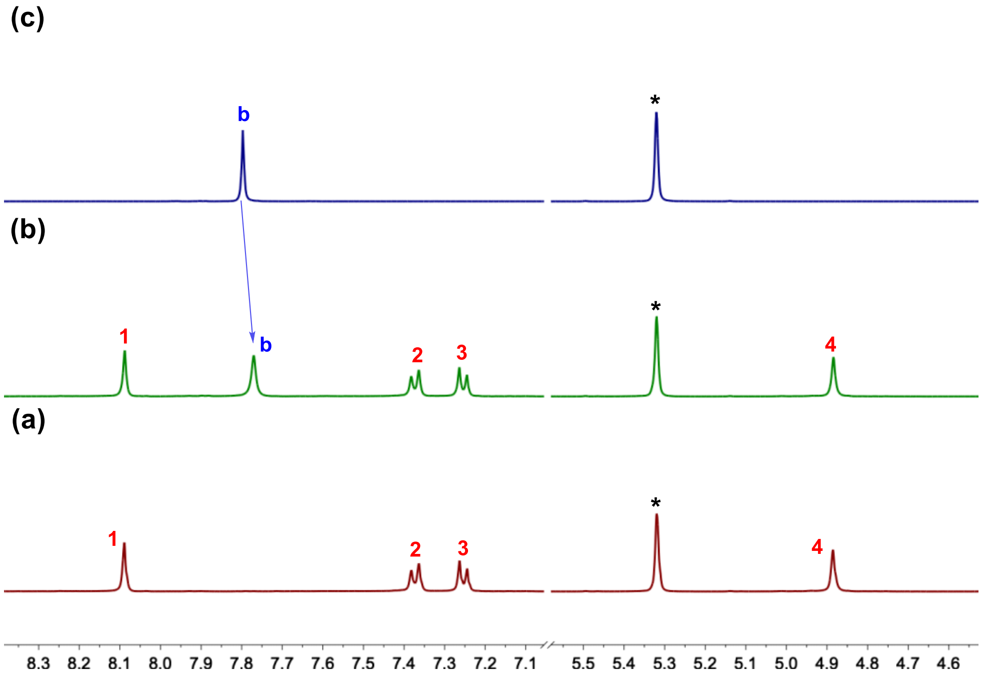

Supplementary Figure 10. Partial ^{1}H NMR spectra (500 MHz, CD_2Cl_2, 298K) of (a) free P4 (5.0 mM), (b) P4 (5.0 mM) and TPN (10.0 mM), and (c) free TPN (5.0 mM).

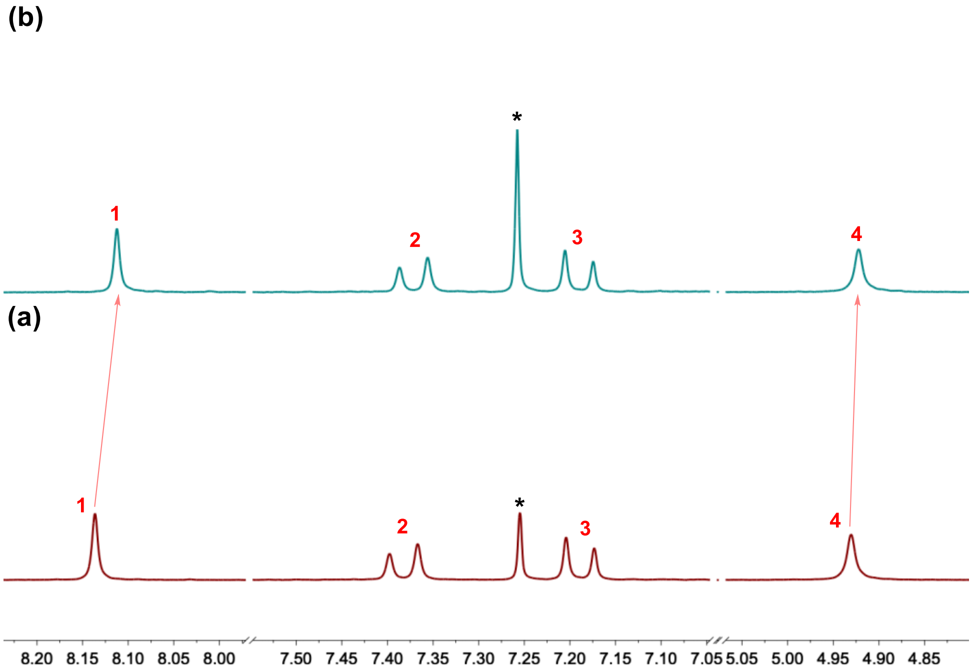

Supplementary Figure 11. Partial ^{1}H NMR spectra (400 MHz, $CDCl_3$, 298K) of (a) free P4 (5.0 mM), (b) P4 (5.0 mM) and TFTN (10.0 mM).

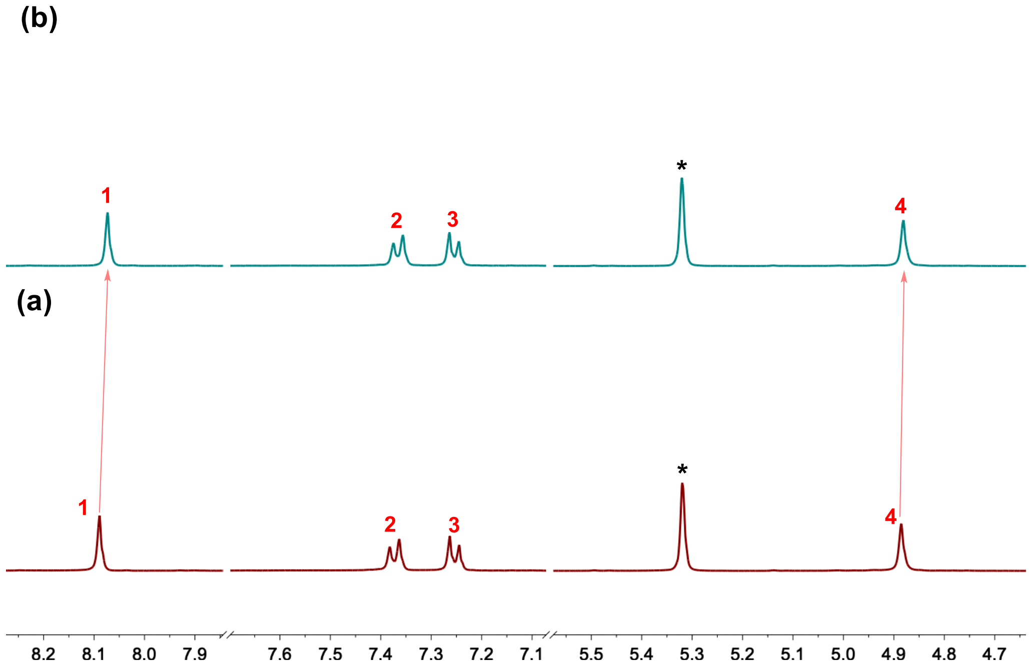

Supplementary Figure 12. Partial ^{1}H NMR spectra (400 MHz, CD_2Cl_2, 298K) of (a) free P4 (5.0 mM), (b) P4 (5.0 mM) and TFTN (10.0 mM).

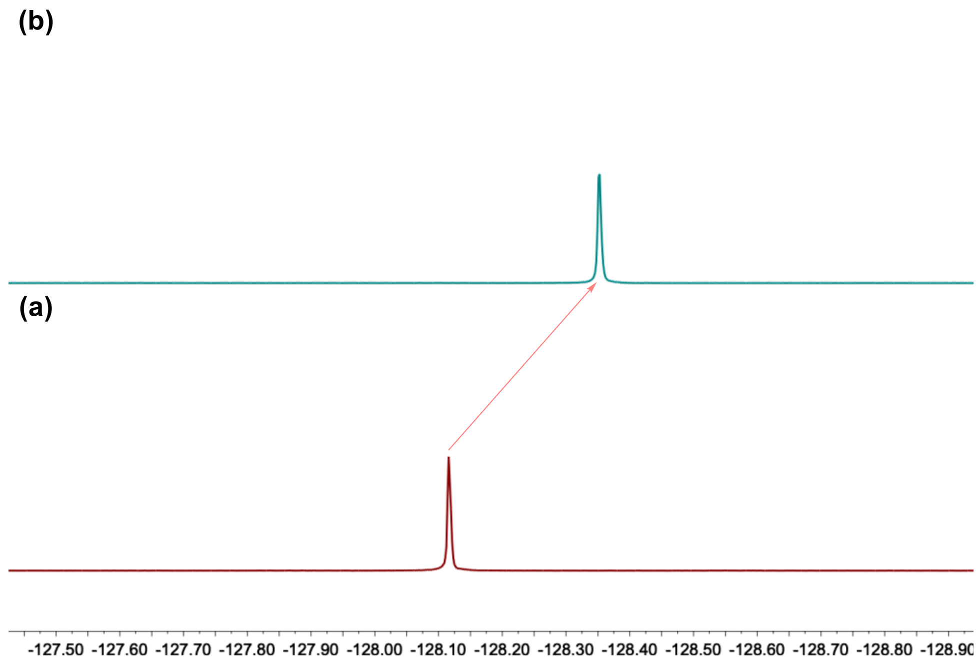

Supplementary Figure 13. Partial ^{19}F NMR spectra (377 MHz, $CDCl_3$, 298K) of (a) free TFTN (5.0 mM), (b) P4 (5.0 mM) and TFTN (10.0 mM).

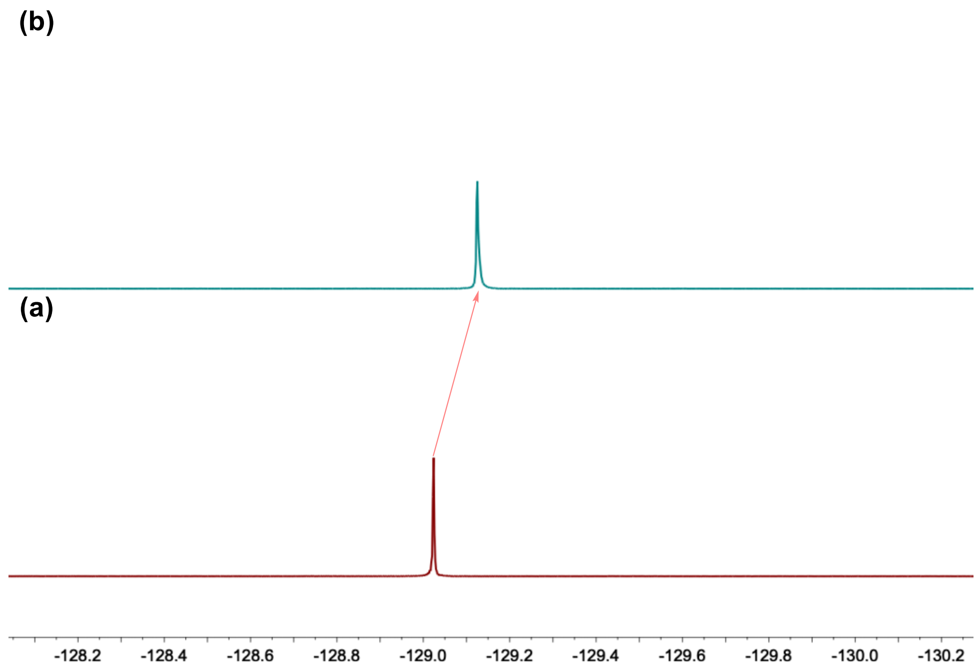

Supplementary Figure 14. Partial ^{19}F NMR spectra (377 MHz, CD_2Cl_2, 298K) of (a) free TFTN (5.0 mM), (b) P4 (5.0 mM) and TFTN (10.0 mM).

2.2 NMR Titration Experiments and Nonlinear Curve Fitting of the Complexes

Supplementary Table 1. Association constants (K_a) for 1:1 complexation of P4 and the electron-deficient guests at 298 K.

Guest	Solvent	K_a [M^{-1}]
DNB	$CDCl_3$	622.7±56.8
DNB	CD_2Cl_2	96.4±15.0
TPN	$CDCl_3$	73.2±12.9
TPN	CD_2Cl_2	[a]
TFTN	$CDCl_3$	[a]
TFTN	CD_2Cl_2	[a]

[a] The association constants were too small to be accurately calculated.

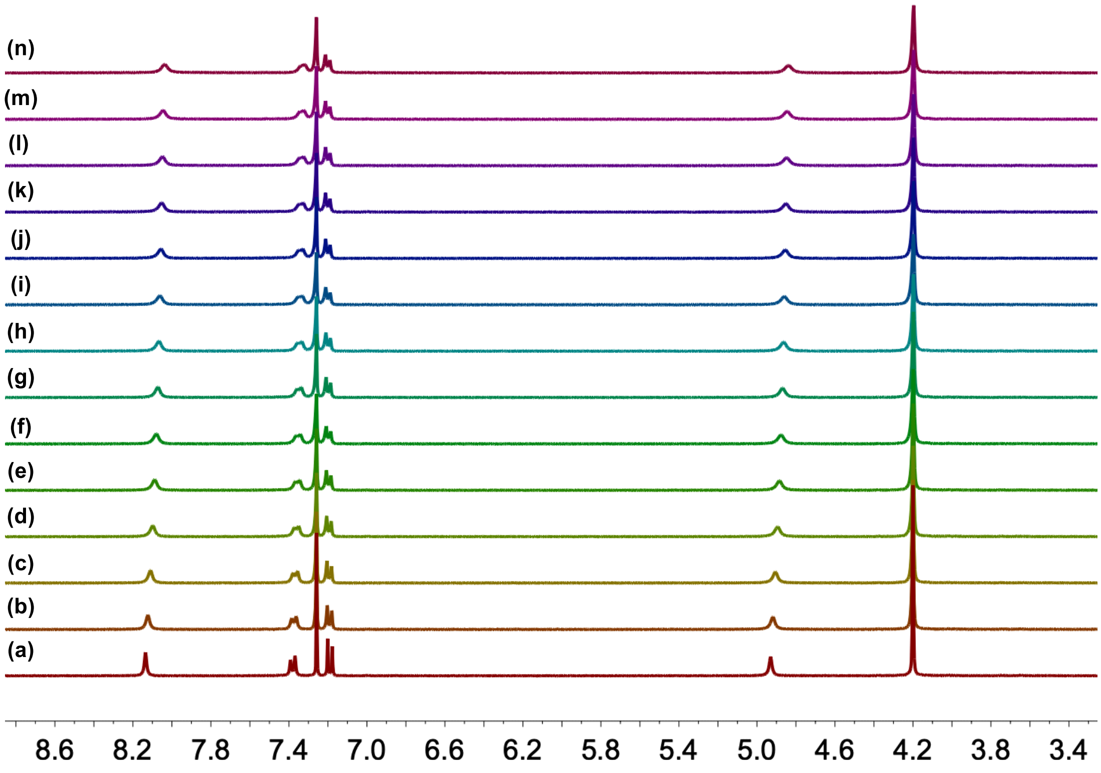

Supplementary Figure 15. Partial ^{1}H NMR spectra (400 MHz, $CDCl_3$, 298 K) of P4 at concentration of 2.0 mM with different concentrations of DNB: (a) 0.0 mM; (b) 0.2 mM; (c) 0.4 mM; (d) 0.6 mM; (e) 0.8 mM; (f) 1.0 mM; (g) 1.2 mM; (h) 1.4 mM; (i) 1.6 mM; (j) 1.8 mM; (k) 2.0 mM; (l) 2.2 mM; (m) 2.4 mM; (n) 3.0 mM.

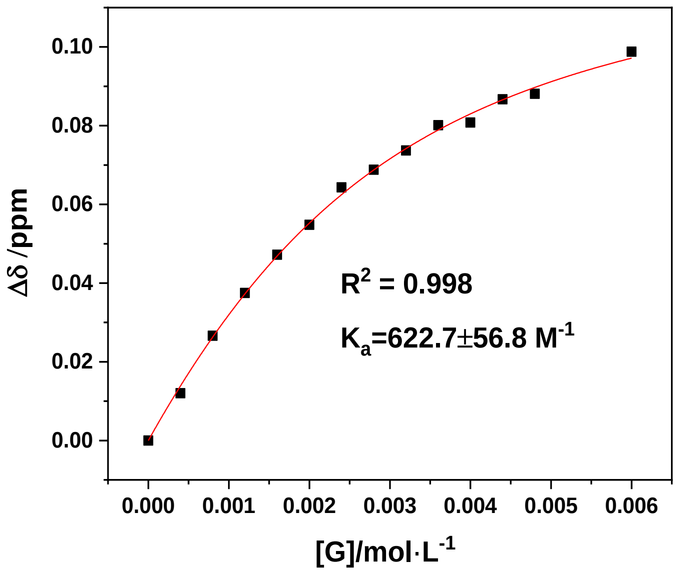

Supplementary Figure 16. Plot of Δδ (ppm) for the H_1 of P4 and DNB in $CDCl_3$ at 298 K.

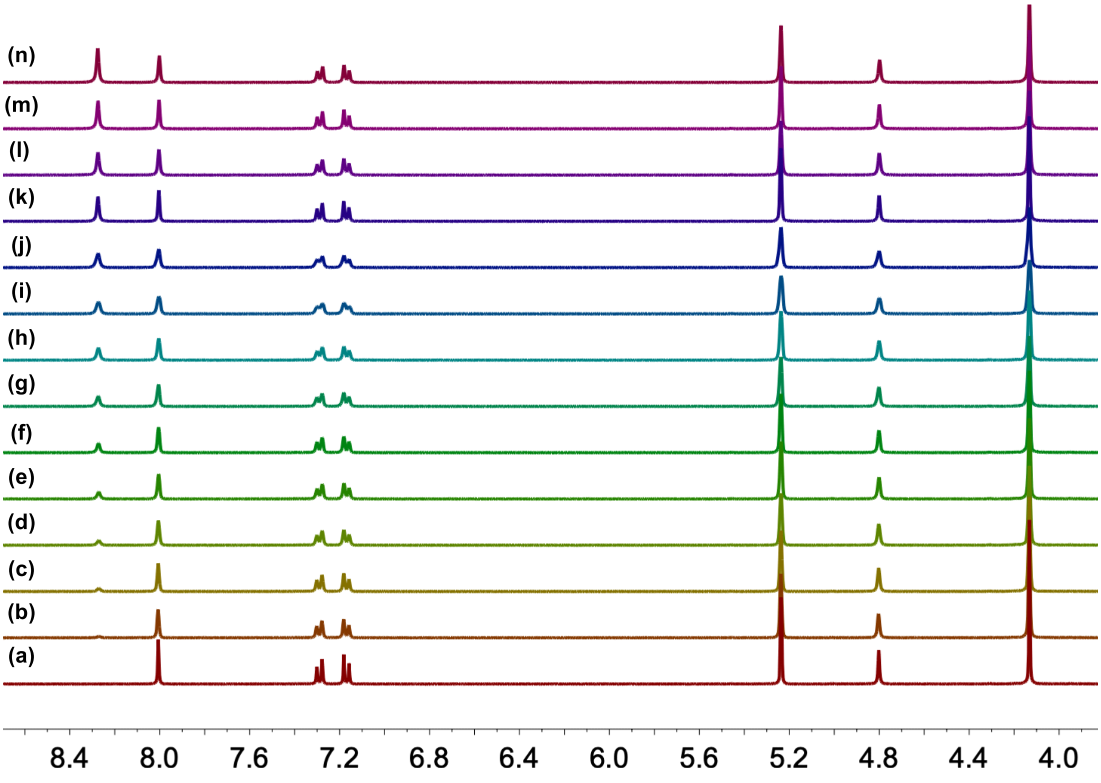

Supplementary Figure 17. Partial ^{1}H NMR spectra (400 MHz, CD_2Cl_2, 298 K) of P4 at concentration of 2.0 mM with different concentrations of DNB: (a) 0.0 mM; (b) 0.2 mM; (c) 0.4 mM; (d) 0.6 mM; (e) 0.8 mM; (f) 1.0 mM; (g) 1.2 mM; (h) 1.4 mM; (i) 1.6 mM; (j) 1.8 mM; (k) 2.0 mM; (l) 2.2 mM; (m) 2.4 mM; (n) 3.0 mM.

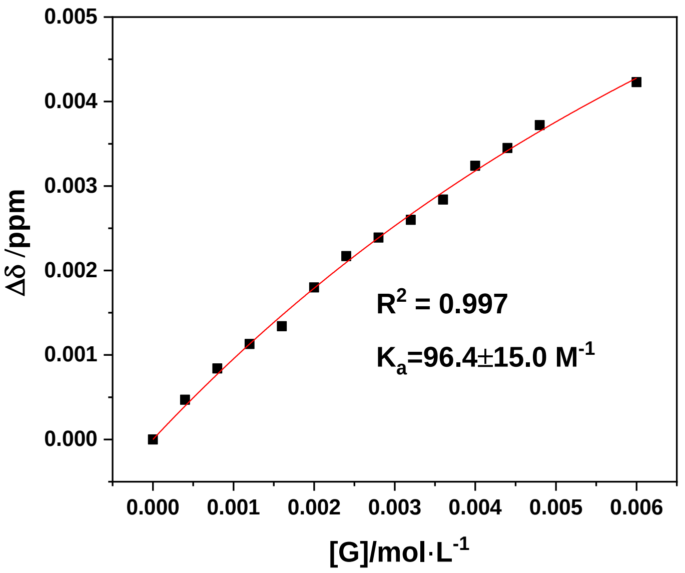

Supplementary Figure 18. Plot of Δδ (ppm) for the H_1 of P4 and DNB in CD_2Cl_2 at 298 K.

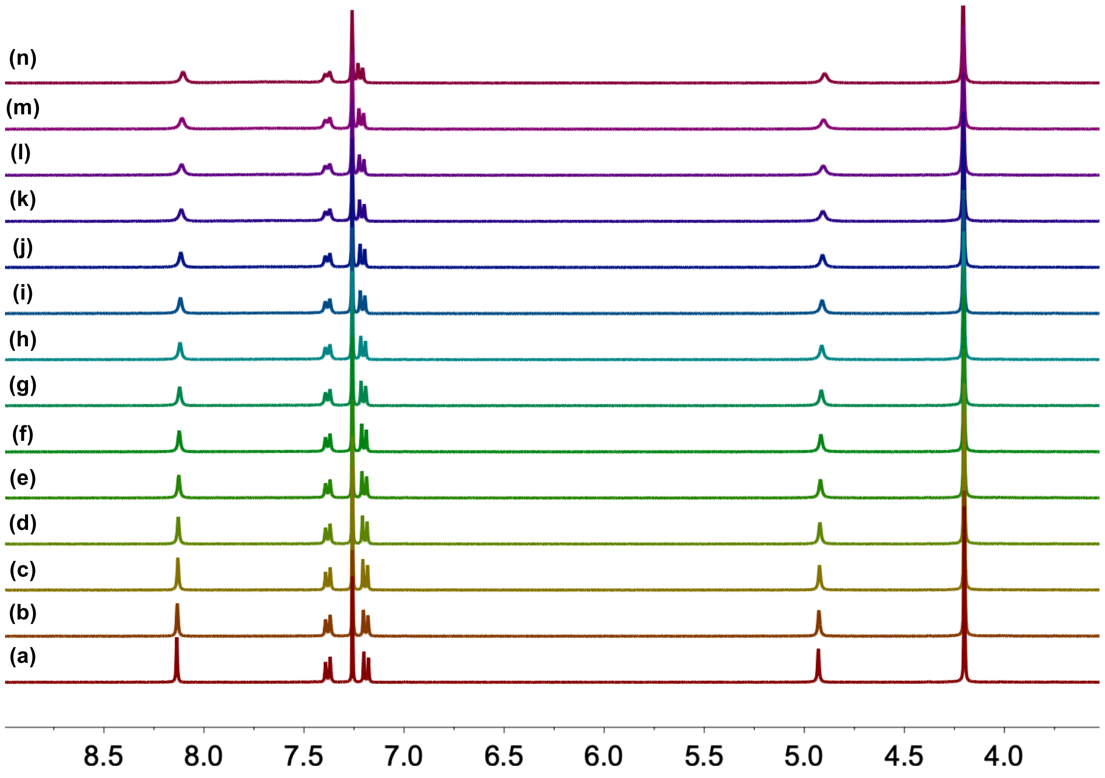

Supplementary Figure 19. Partial ^{1}H NMR spectra (400 MHz, $CDCl_3$, 298 K) of P4 at concentration of 2.0 mM with different concentrations of TPN: (a) 0.0 mM; (b) 0.2 mM; (c) 0.4 mM; (d) 0.6 mM; (e) 0.8 mM; (f) 1.0 mM; (g) 1.2 mM; (h) 1.4 mM; (i) 1.6 mM; (j) 1.8 mM; (k) 2.0 mM; (l) 2.2 mM; (m) 2.4 mM; (n) 3.0 mM.

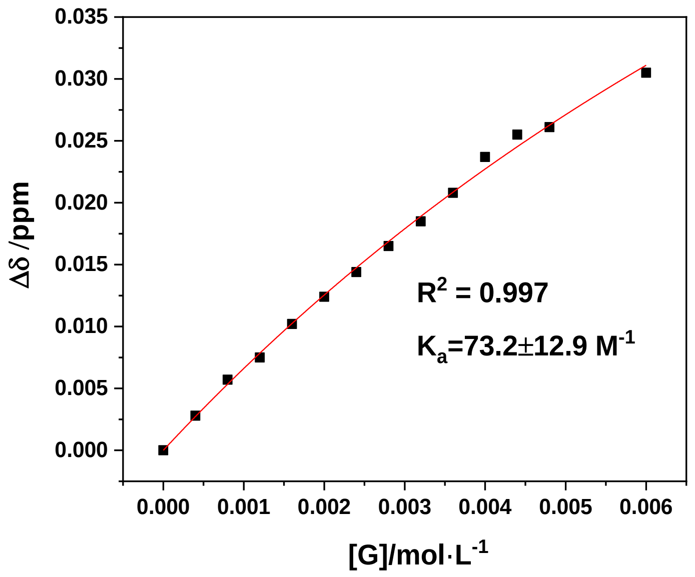

Supplementary Figure 20. Plot of Δδ (ppm) for the H_1 of P4 and TPN in $CDCl_3$ at 298 K.

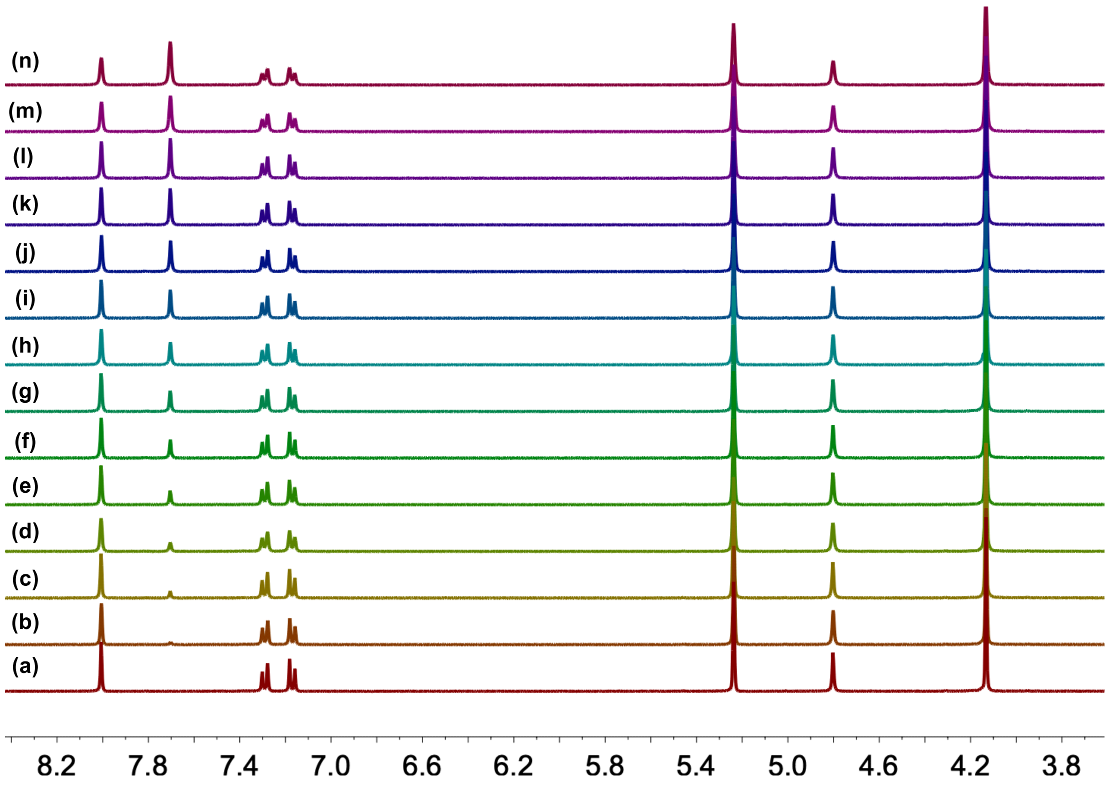

Supplementary Figure 21. Partial ^1H NMR spectra (400 MHz, CD_2Cl_2, 298 K) of P4 at concentration of 2.0 mM with different concentrations of TPN: (a) 0.0 mM; (b) 0.2 mM; (c) 0.4 mM; (d) 0.6 mM; (e) 0.8 mM; (f) 1.0 mM; (g) 1.2 mM; (h) 1.4 mM; (i) 1.6 mM; (j) 1.8 mM; (k) 2.0 mM; (l) 2.2 mM; (m) 2.4 mM; (n) 3.0 mM.

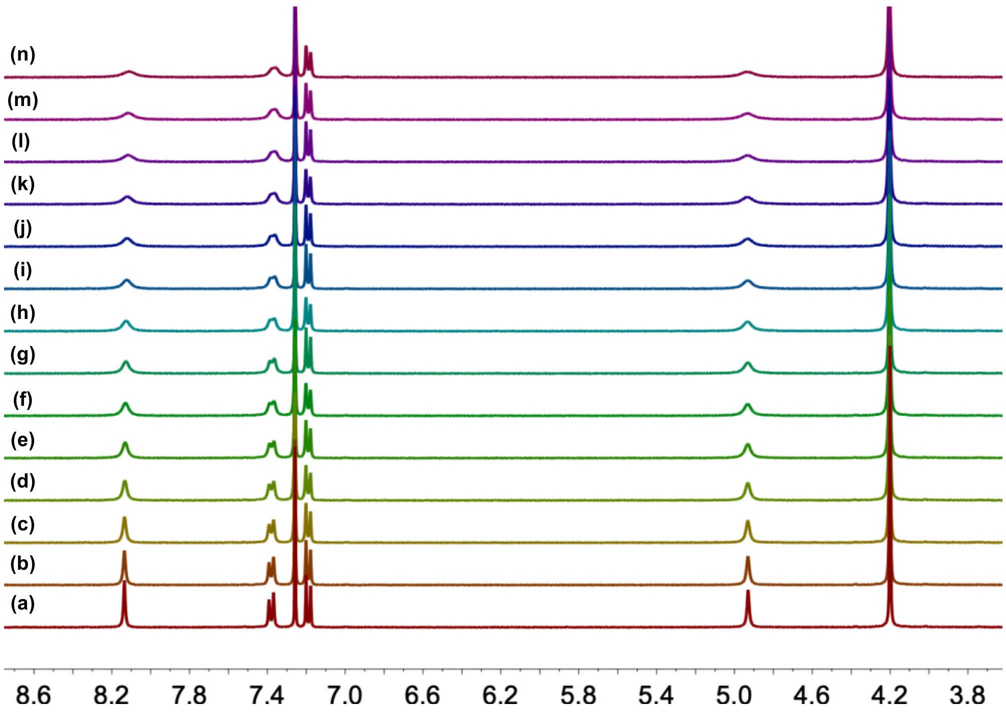

Supplementary Figure 22. Partial ^{1}H NMR spectra (400 MHz, $CDCl_3$, 298 K) of P4 at concentration of 2.0 mM with different concentrations of TPN: (a) 0.0 mM; (b) 0.2 mM; (c) 0.4 mM; (d) 0.6 mM; (e) 0.8 mM; (f) 1.0 mM; (g) 1.2 mM; (h) 1.4 mM; (i) 1.6 mM; (j) 1.8 mM; (k) 2.0 mM; (l) 2.2 mM; (m) 2.4 mM; (n) 3.0 mM.

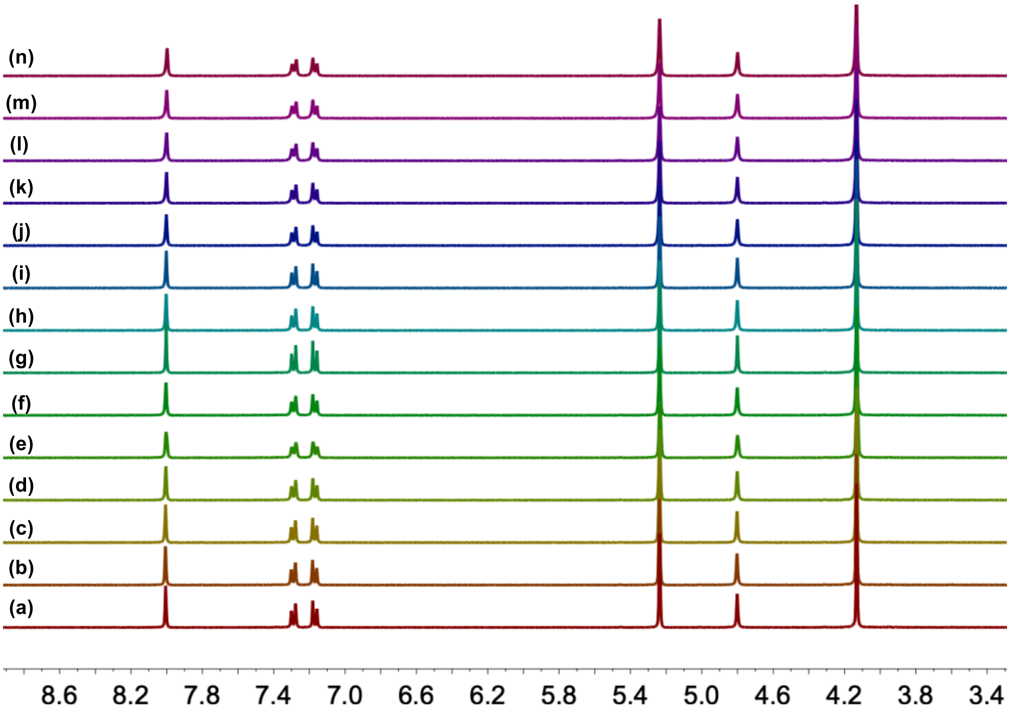

Supplementary Figure 23. Partial ^{1}H NMR spectra (400 MHz, CD_2Cl_2, 298 K) of P4 at concentration of 2.0 mM with different concentrations of TPN: (a) 0.0 mM; (b) 0.2 mM; (c) 0.4 mM; (d) 0.6 mM; (e) 0.8 mM; (f) 1.0 mM; (g) 1.2 mM; (h) 1.4 mM; (i) 1.6 mM; (j) 1.8 mM; (k) 2.0 mM; (l) 2.2 mM; (m) 2.4 mM; (n) 3.0 mM.

3. UV-vis Absorption Spectra of P4 with TPN and TFTN

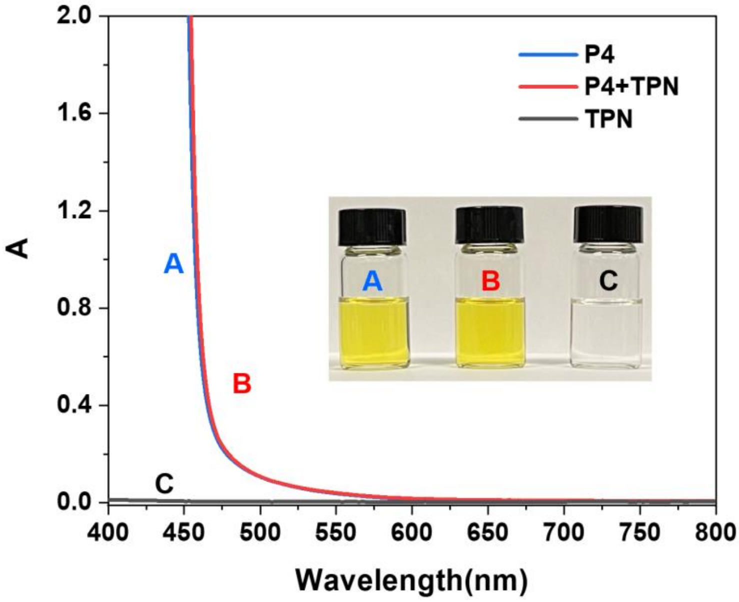

Supplementary Figure 24. UV-vis spectra ($CHCl_3$): A, P4 (3.0 mM); B, P4 (3.0 mM) and TPN (6.0 mM); C, TPN (6.0 mM). The inserted optical image shows the solution colors.

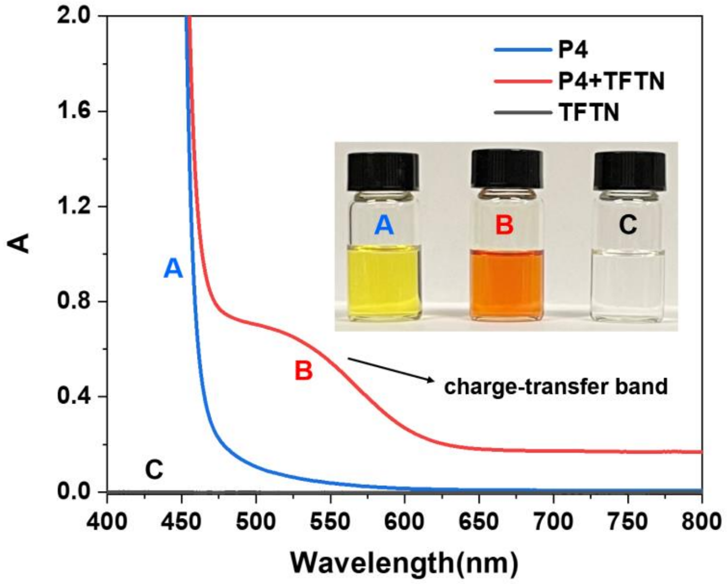

Supplementary Figure 25. UV-vis spectra ($CHCl_3$): A, P4 (3.0 mM); B, P4 (3.0 mM) and TFTN (6.0 mM); C, TFTN (6.0 mM). The inserted optical image shows the solution color change by charge-transfer interaction between P4 and TFTN.

4. Crystal Engineering of Pagoda[4]arene Crystals

4.1. Structure of n-Hexane@P4

Method. Slow diffusion of *n*-hexane to a solution of P4 in CH_2Cl_2 afforded yellow block crystals suitable for X-ray diffraction. Using Olex2, the structure was refined with the ShelXL refinement package using Least Squares minimization.

Crystal Data. Empirical formula = [($C_{77}H_{77}O_8$)], formula weight = 1130.38, crystal system = monoclinic, space group = $P2_1/n$, a = 11.8713(2) Å, b = 21.3316(3) Å, c = 24.2532(4) Å, α = 90°, β = 100.2060(10)°, γ = 90°, V = 6044.55(17) Å^3, Z = 4, T = 169.98(16) K, μ(CuKα) = 0.622 mm^{-1}, D_{calc} = 1.242 g/cm^3, 36134 reflections measured (5.556 ≤ 2Θ ≤ 151.028), 11722 unique (R_{int} = 0.0339, R_{sigma} = 0.0345) which were used in all calculations. The final R_1 was 0.0637 ($I > 2\sigma(I)$) and wR_2 was 0.2054 (all data). CCDC number: 1975851

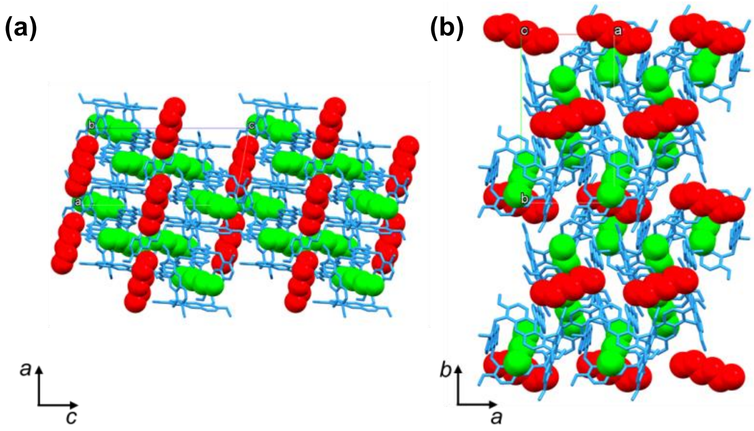

Supplementary Figure 26. Packing mode of *n*-hexane@P4 viewed along (a) *b*-axis and (b) *c*-axis showing the absence of the in plane 2D π···π tiling pattern. Different colors represent the symmetry equivalence and hydrogen atoms are omitted for the sake of clarity.

4.2. Structure of CH_2Cl_2@P4

Method. Slow diffusion of methanol to a solution of P4 in CH_2Cl_2 afforded yellow cube crystals suitable for X-ray diffraction.

Crystal Data. Empirical formula = [($C_{69}H_{58}Cl_2O_8$)], formula weight = 1086.05, crystal system = monoclinic, space group = $I2/a$, a = 15.7416(5) Å, b = 18.3691(6) Å, c = 18.8001(6) Å, α = 90°, β = 98.056(3)°, γ = 90°, V = 5382.5(3) Å^3, Z = 4, T = 170.00(15) K, μ(CuKα) = 1.572 mm^{-1}, D_{calc} = 1.340 g/cm^3, 19285 reflections measured (6.76 ≤ 2Θ ≤ 150.844), 5291 unique (R_{int} = 0.0763, R_{sigma} = 0.0660) which were used in all calculations. The final R_1 was 0.0853 (I > 2σ(I)) and wR_2 was 0.2763 (all data). CCDC number: 1975860.

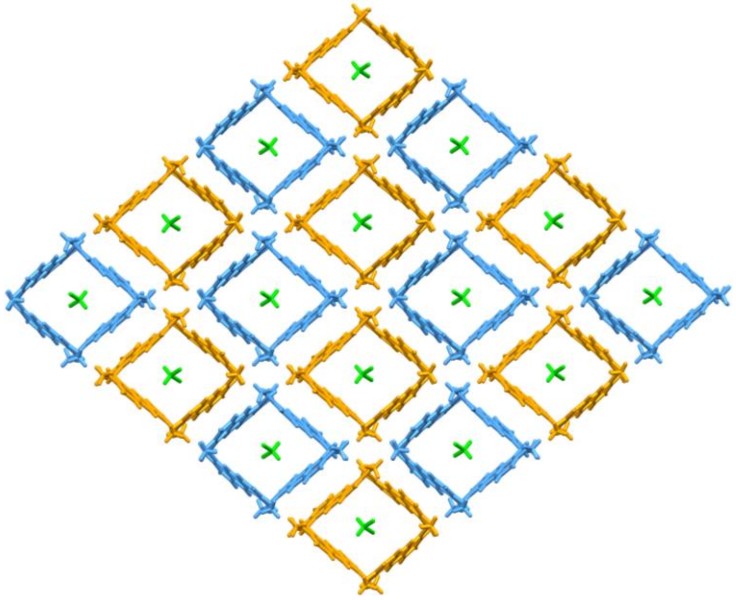

Supplementary Figure 27. Two-dimensional regular rhombic tiling in a plane showing the enantiomers of P4 arranged alternately. P4 in color blue represent the crystal structures of *pS*-P4 and the orange color P4 represent *pR*-P4.

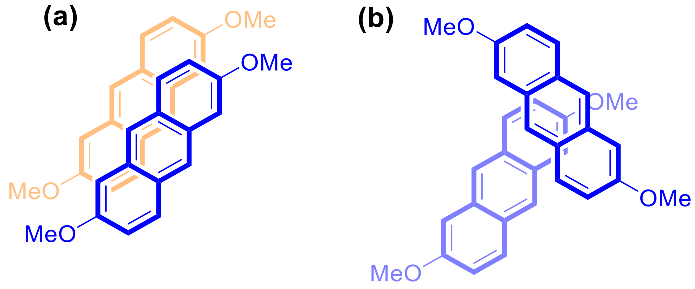

Supplementary Figure 28. Schematic representations of two types of π-π stacking: (a) parallel face-to-face manner and (b) off-set (staggered) face-to-face manner.

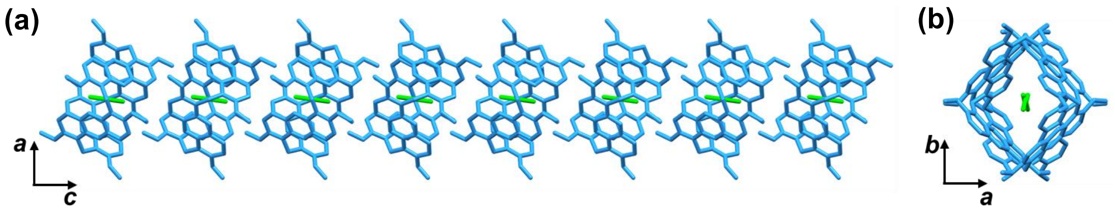

Supplementary Figure 29. Packing of P4 units in crystal CH_2Cl_2@P4 along (a) *b*-axis and (b) *c*-axis. The colors represent the symmetry equivalence and hydrogen atoms are removed for the sake of clarity.

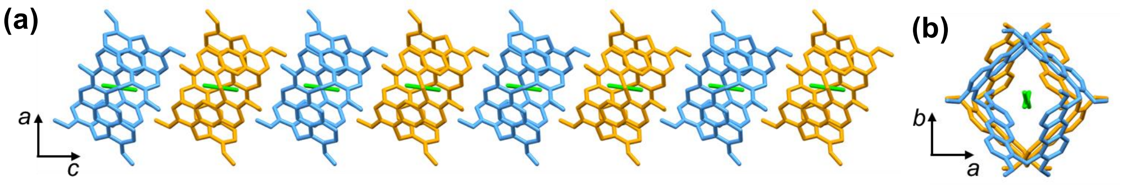

Supplementary Figure 30. Packing of P4 units in crystal CH_2Cl_2@P4 along (a) *b*-axis and (b) *c*-axis. Hydrogen atoms are removed for the sake of clarity. P4 in color blue represent the crystal structures of *pS*-P4 and the orange color P4 represent *pR*-P4.

5. Crystal Engineering of Pagoda[4]arene-Based Co-crystals

5.1 Crystal Structure of P4-DNB$_a$

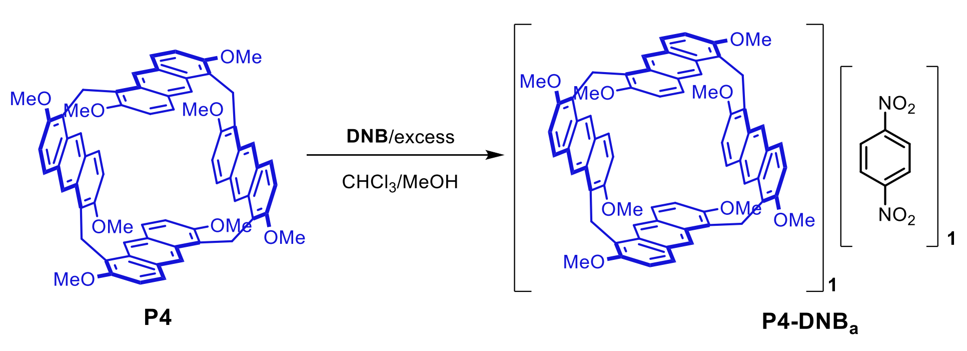

Supplementary Figure 31. Co-crystallization of P4 with DNB to afford P4-DNB$_a$.

Method. Dark brown block crystals were obtained by MeOH vapor diffusion into a 2 mL $CHCl_3$ solution containing P4 (5 mg) and DNB (4 mg). The crystals were isolated for single crystal X-ray diffraction. The molar ratio of the P4 and DNB in the crystal structure was 1 : 1.

Crystal Data. Empirical formula = [($C_{75}H_{61}Cl_3N_2O_{12}$)], formula weight = 1288.60, crystal system = monoclinic, space group = $P2_1/n$, a = 23.17880(10) Å, b = 22.80780(10) Å, c = 25.69640(10) Å, α = 90°, β = 104.1010(10)°, γ = 90°, V = 13175.26(11) Å^3, Z = 8, T = 169.99(10) K, μ(CuKα) = 1.791 mm^{-1}, D_{calc} = 1.299 g/cm^3, 104377 reflections measured (4.608 ≤ 2Θ ≤ 150.842), 26247 unique (R_{int} = 0.0267, R_{sigma} = 0.0219) which were used in all calculations. The final R_1 was 0.0864 (I > 2σ(I)) and wR_2 was 0.2408 (all data). CCDC number: 2087963

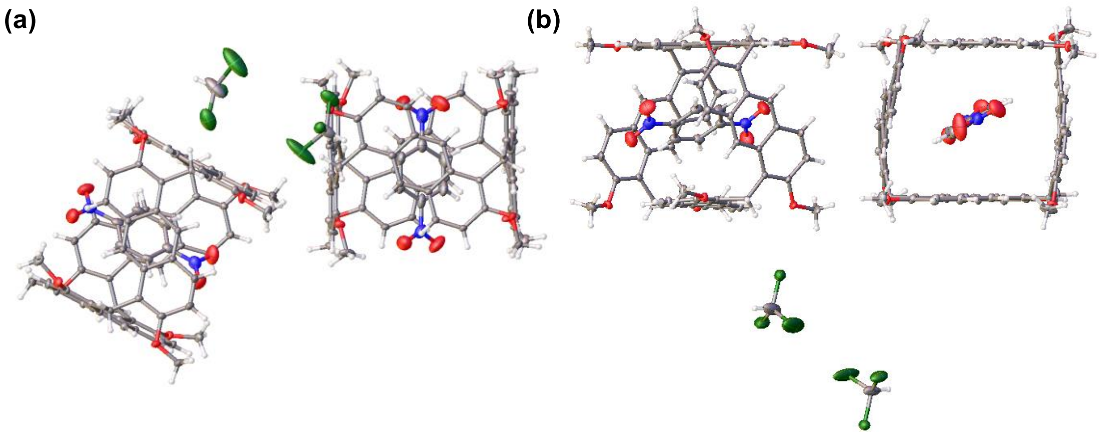

Supplementary Figure 32. ORTEP drawing of P4-DNB$_a$ from (a) side view and (b) top view (the thermal ellipsoids are displayed at a 30 % probability).

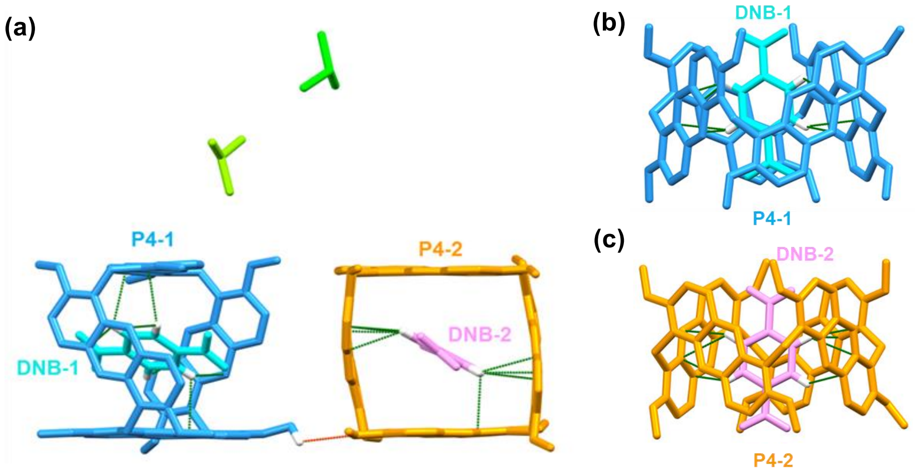

Supplementary Figure 33. (a) Crystal structure of P4-DNB$_a$ showing the asymmetric unit containing two enantiomers P4, two DNB molecules and two $CHCl_3$ molecules from the top view. Sop view of crystal structure showing the interactions between (b) DNB-1 and P4-1 and (c) DNB-2 and P4-2. Different colors represent the symmetry equivalence. Hydrogen atoms (in white color) not involved in the noncovalent interactions are omitted for clarity. Green and red dotted line represent the noncovalent interactions.

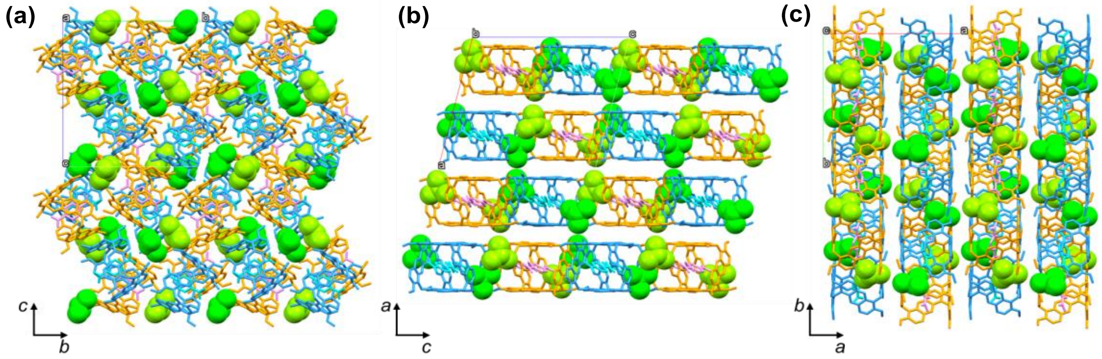

Supplementary Figure 34. Packing mode of P4-DNB$_a$ viewed along (a) *a*-axis (b) *b*-axis and (c) *c*-axis showing the absence of the in plane 2D tiling pattern. Different colors represent the symmetry equivalence and hydrogen atoms are omitted for the sake of clarity.

5.2 Crystal Structure of P4-DNB$_b$

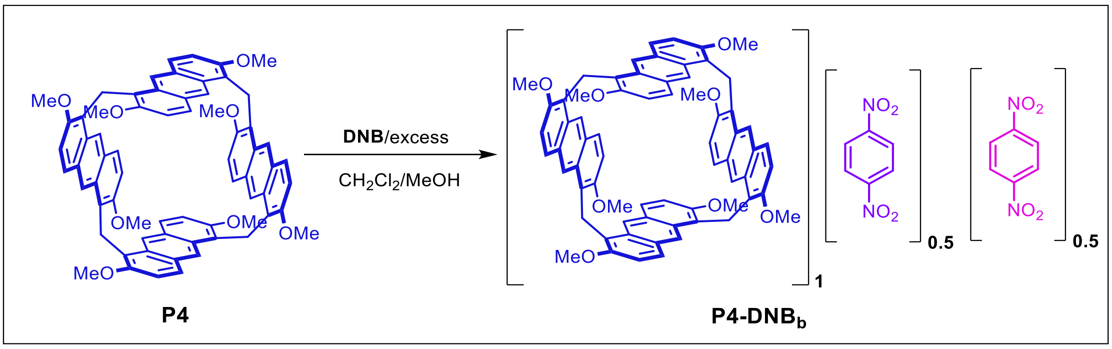

Supplementary Figure 35. Co-crystallization of P4 with DNB to afford P4-DNB$_b$.

Method. Dark brown block crystals were obtained by MeOH vapor diffusion into a 2 mL CH_2Cl_2 solution containing P4 (5 mg) and DNB (4 mg). The crystals were isolated for single crystal X-ray diffraction. The molar ratio of the P4 and DNB in the crystal structure was 1 : 1.

Crystal Data. Empirical formula = [($C_{76}H_{64}Cl_4N_2O_{12}$)], formula weight = 1339.09, crystal system = triclinic, space group = *P*-1, *a* = 13.6989(4) Å, *b* = 13.7879(4) Å, *c* =

17.7478(6) Å, α = 110.554(3)°, β = 94.114(3)°, γ = 90.114(3)°, V = 3129.38(18) $Å^3$, Z = 2, T = 169.99(11) K, μ(CuKα) = 2.290 mm^{-1}, D_{calc} = 1.421 g/cm^3, 43262 reflections measured (5.334 ≤ 2Θ ≤ 151.102), 12431 unique (R_{int} = 0.0413, R_{sigma} = 0.0338) which were used in all calculations. The final R_1 was 0.1047 (I > 2σ(I)) and wR_2 was 0.3482 (all data). CCDC number: 2087964.

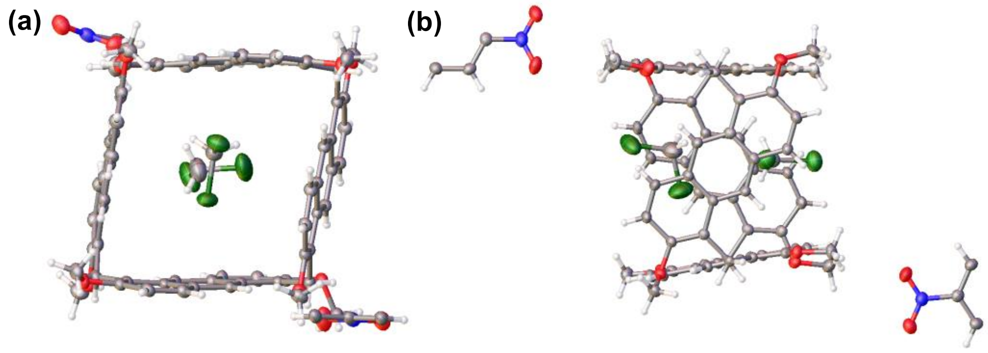

Supplementary Figure 36. ORTEP drawing of P4-DNB$_b$ from (a) top view and (b) side view (the thermal ellipsoids are displayed at a 30 % probability).

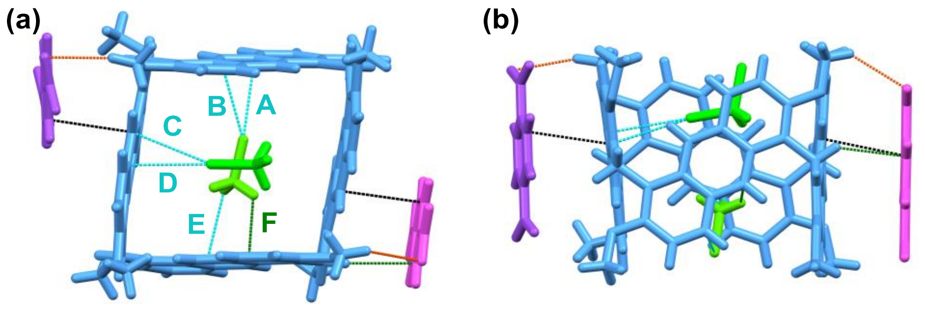

Supplementary Figure 37. Crystal structure of P4-DNB$_b$ showing two CH_2Cl_2 molecules were encapsulated in the cavity of P4 by Cl···π and C-H···π interactions from (a) top view and (b) side view. Blue and green dashed lines indicate Cl···π interactions (A-E) and C-H···π interactions (F). Detailed parameters are as follows. Cl···π distances (Angstroms): A, 3.25; B, 3.43; C, 3.41; D, 3.27; E, 3,36. C-H···π distances (Angstroms): F, 2.84. C-H···π angles (degrees): F, 123.82.

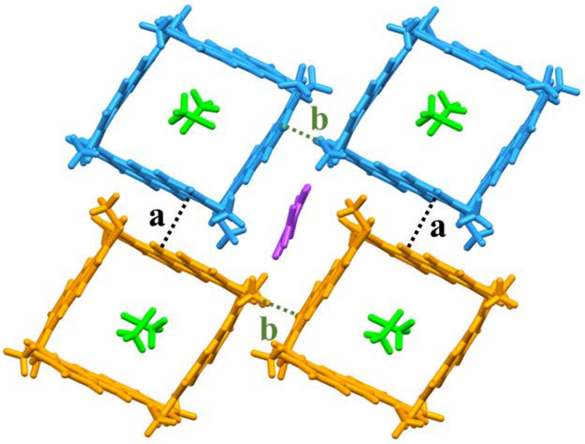

Supplementary Figure 38. Crystal structure of P4-DNB$_b$ showing the different noncovalent interactions between adjacent P4. P4 in color blue represent the crystal structures of *pS*-P4 and the orange color P4 represent *pR*-P4.

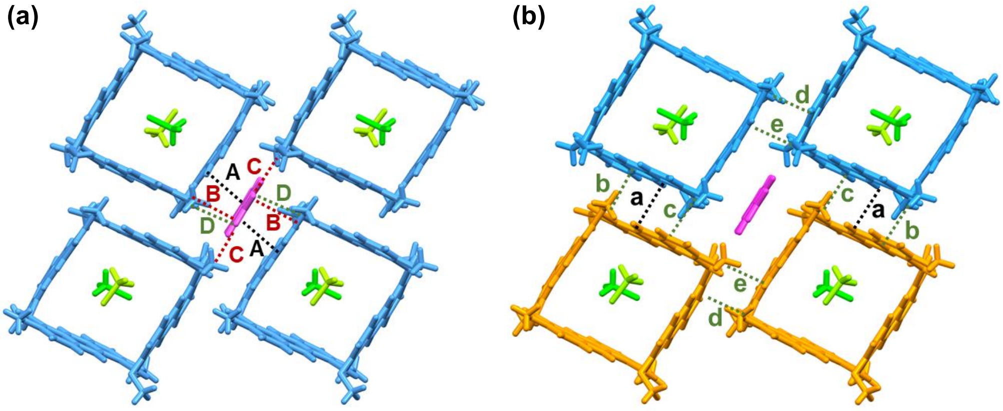

Supplementary Figure 39. Crystal structure of P4-DNB$_b$ showing (a) the parallelogram tiling unit formed by one DNB-2 and four P4 around through CT and multiple noncovalent interactions. (b) the different noncovalent interactions between adjacent P4. P4 in color blue represent the crystal structures of *pS*-P4 and the orange color P4 represent *pR*-P4. Black, red and green dashed lines represent π···π interactions (A and a), C-H···O interactions (B and C) and C-H···π interactions (D, b, c, d and e), respectively. Detailed parameters are as follows. The centroid-plane distances (Angstroms): A, 3.39; a, 3.49, and the corresponding dihedral angles (degrees): A, 1.89; a, 0. C-H···O distances (Angstroms): B, 2.68; C, 2.59. C-H···O angles (degrees): B, 133.56; C, 146.07. C-H···π distances (Angstroms): D, 2.81; b, 2.84; c, 2.84; d, 2.89 and e, 2.88. C-H···π angles (degrees): D, 138.23; b, 151.92; c, 151.92 d, 141.59 and e,

135.85.

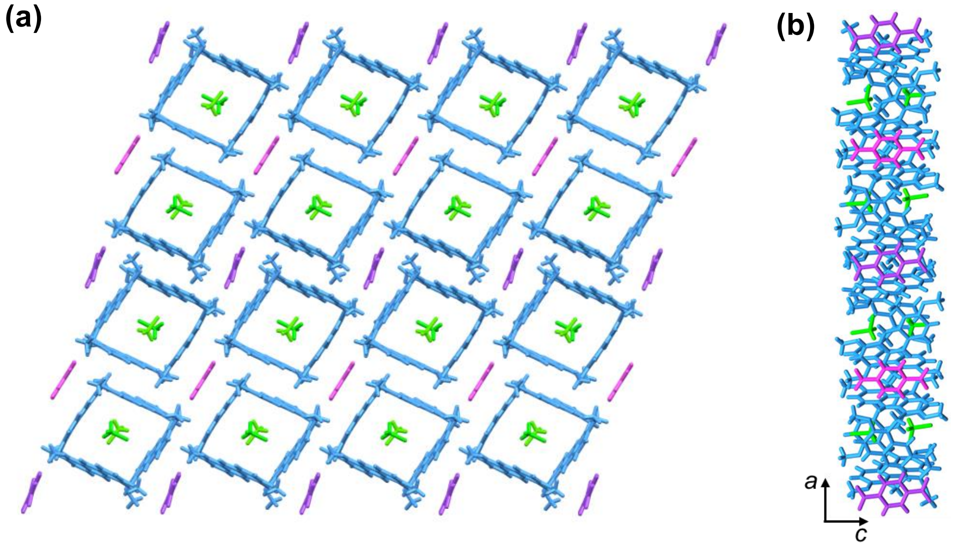

Supplementary Figure 40. Crystal structures of the layer-like superstructure of P4-DNB$_b$ (a) from the top view and (b) from side view in the *ac*-plane. The colors represent the symmetry equivalence.

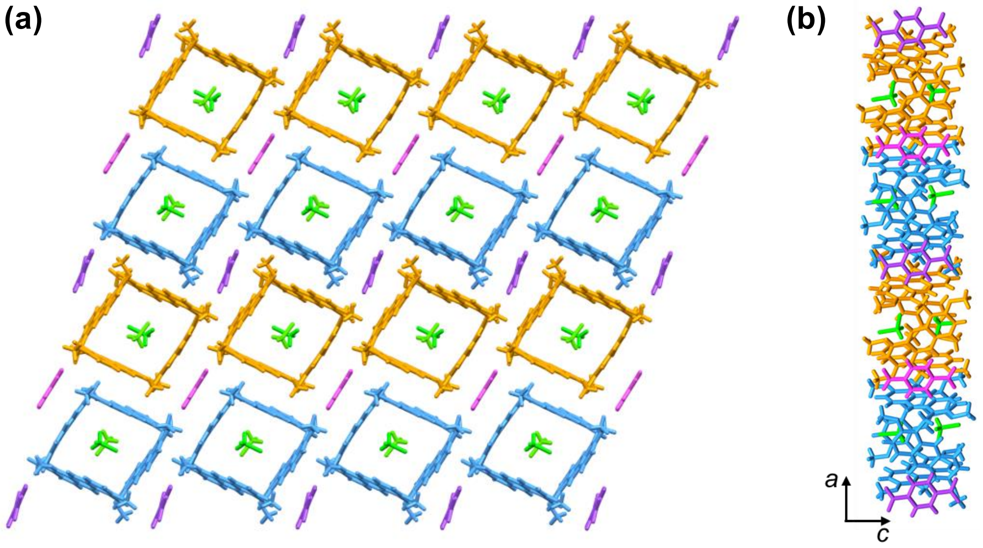

Supplementary Figure 41. Crystal structures of the layer-like superstructure of P4-DNB$_b$ (a) from the top view and (b) from side view in the *ac*-plane. The colors represent the symmetry equivalence except that P4 in color blue represent the crystal structures of *pS*-P4 and the orange color P4 represent *pR*-P4.

5.3 Crystal Structure of P4-TPN$_a$

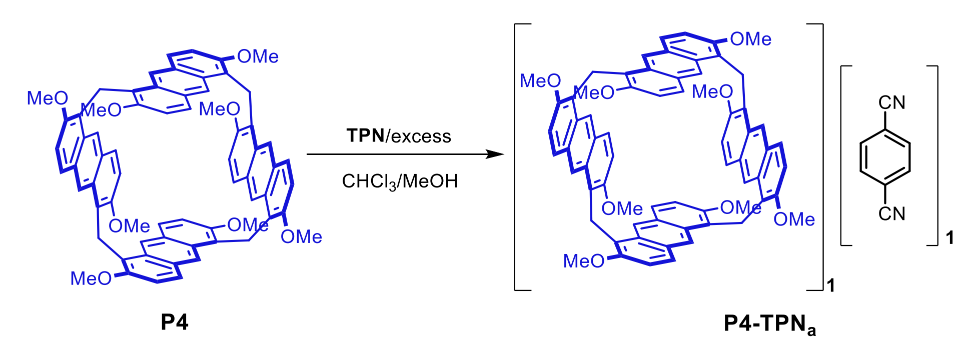

Supplementary Figure 42. Co-crystallization of P4 with TPN to afford P4-TPN$_a$.

Method. Yellow crystals were obtained by MeOH vapor diffusion into a 2 mL $CHCl_3$ solution containing P4 (5 mg) and TPN (3 mg). The crystals were isolated for single crystal X-ray diffraction. The molar ratio of the P4 and TPN in the crystal structure was 1 : 1.

Crystal Data. Empirical formula = [($C_{77}H_{61}Cl_3N_2O_8$)], formula weight = 1248.62, crystal system = monoclinic, space group = $P2_1/n$, a = 23.2353(7) Å, b = 22.7247(5) Å, c = 25.7071(6) Å, α = 90 °, β = 105.340(3) °, γ = 90 °, V = 13090.1(6) Å^3, Z = 8, T = 169.99(14) K, μ(CuKα) = 1.739 mm^{-1}, D_{calc} = 1.267 g/cm^3, 22670 reflections measured (4.562 ≤ 2Θ ≤ 132), 22670 unique (R_{int} = 0.0923, R_{sigma} = 0.0641) which were used in all calculations. The final R_1 was 0.1776 (I > 2σ(I)) and wR_2 was 0.1776 (all data). CCDC number: 2087965.

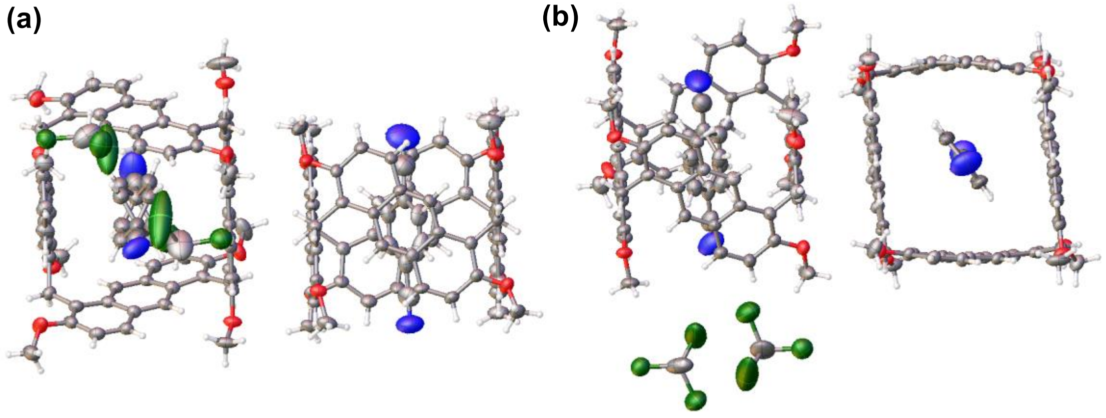

Supplementary Figure 43. ORTEP drawing of P4-TPN$_a$ from (a) side view and (b) top view (the thermal ellipsoids are displayed at a 30 % probability).

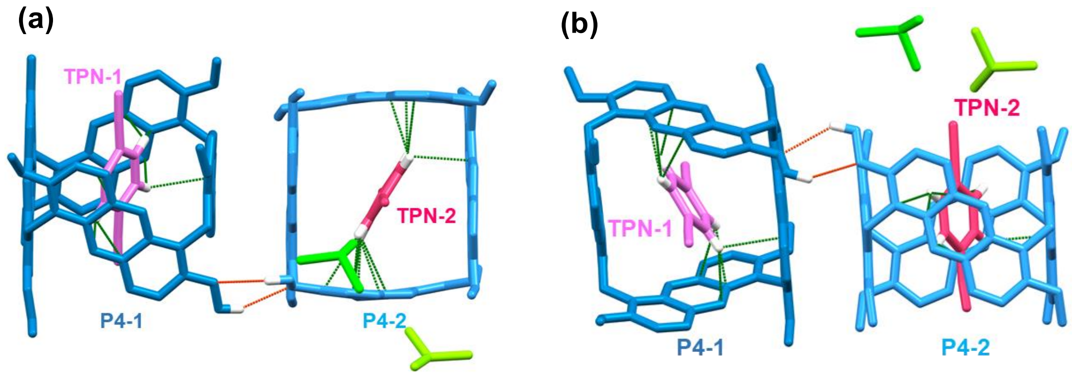

Supplementary Figure 44. Crystal structure of P4-TPN$_a$ showing the asymmetric unit containing two enantiomers P4, two TPN molecules and two $CHCl_3$ molecules from (a) top view and (b) side view. Different colors showing the symmetry equivalence. Hydrogen atoms (in white color) not involved in the noncovalent interactions are omitted for clarity. Green dotted lines represent C-H···π interactions and red dotted lines represent the C-H···O interactions with distances of 2.59 and 2.50 Å between two P4.

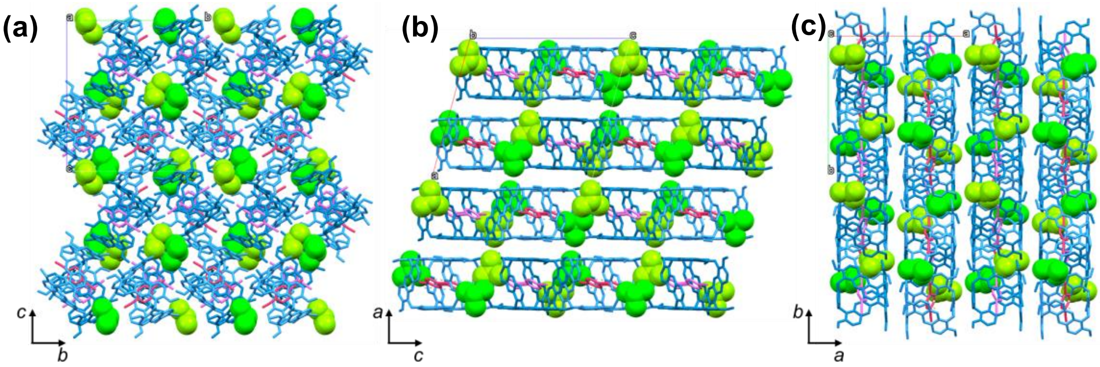

Supplementary Figure 45. Packing mode of P4-TPN_a viewed along (a) *a*-axis (b) *b*-axis and (c) *c*-axis showing the absence of the in plane 2D tiling pattern. Different colors represent the symmetry equivalence and hydrogen atoms are omitted for the sake of clarity.

5.4 Crystal Structure of P4-TPN_b

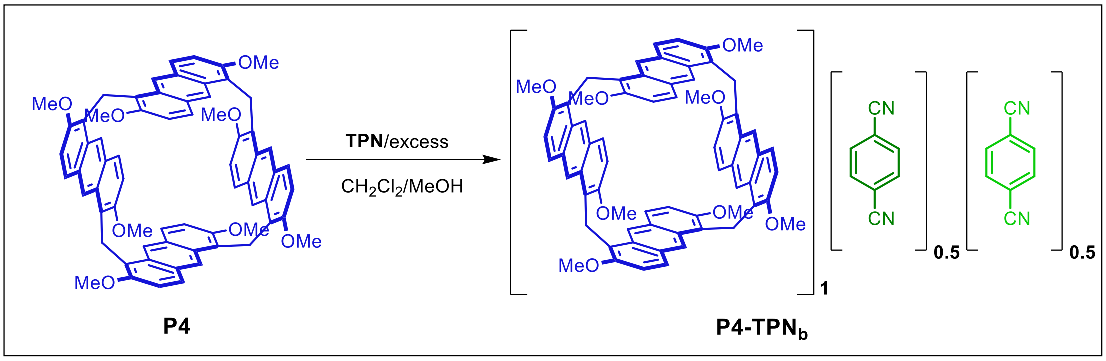

Supplementary Figure 46. Co-crystallization of P4 with TPN to afford P4-TPN_b.

Method. Yellow crystals were obtained by MeOH vapor diffusion into a 2 mL CH_2Cl_2 solution containing P4 (5 mg) and TPN (3 mg). The crystals were isolated for single crystal X-ray diffraction. The molar ratio of the P4 and TPN in the crystal structure was 1 : 1.

Crystal Data. Empirical formula = [($C_{78}H_{64}Cl_4N_2O_8$)], formula weight = 1299.11, crystal system = triclinic, space group = *P*-1, a = 13.6509(4) Å, b = 13.8005(4) Å, c = 17.8041(4) Å, α = 69.609(3) °, β = 86.172(2) °, γ = 89.886(2) °, V = 3136.10(16) Å^3, Z

= 2, T = 169.99(13) K, μ(CuKα) = 2.219 mm^{-1}, D_{calc} = 1.376 g/cm^3, 32396 reflections measured (5.308 ≤ 2Θ ≤ 150.902), 12130 unique (R_{int} = 0.0417, R_{sigma} = 0.0417) which were used in all calculations. The final R_1 was 0.1114 (I > 2σ(I)) and wR_2 was 0.2899 (all data). CCDC number: 2087966.

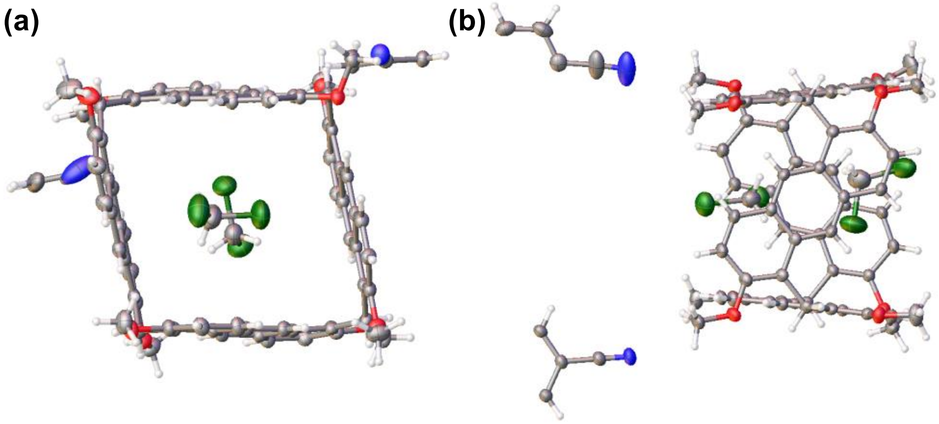

Supplementary Figure 47. ORTEP drawing of P4-TPN$_b$ from (a) top view and (b) side view (the thermal ellipsoids are displayed at a 30 % probability).

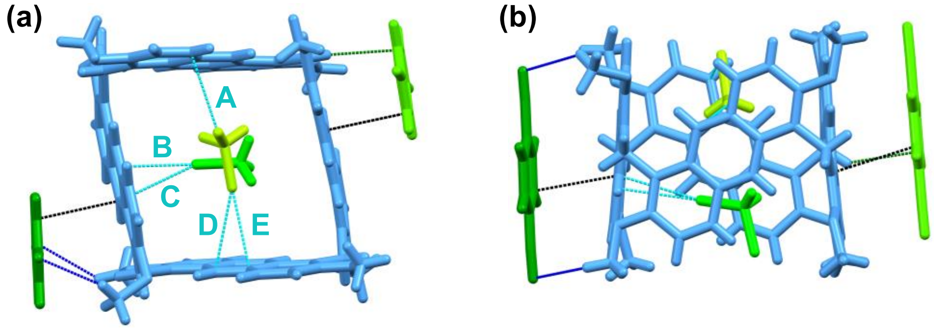

Supplementary Figure 48. Crystal structure of P4-TPN$_b$ showing two CH_2Cl_2 molecules were encapsulated in the cavity of P4 by Cl···π interactions from (a) top view and (b) side view. Blue dashed lines indicate Cl···π interactions (A-E). Detailed parameters are as follows. Cl···π distances (Angstroms): A, 3.35; B, 3.26; C, 3.38; D, 3.38; E, 3,26.

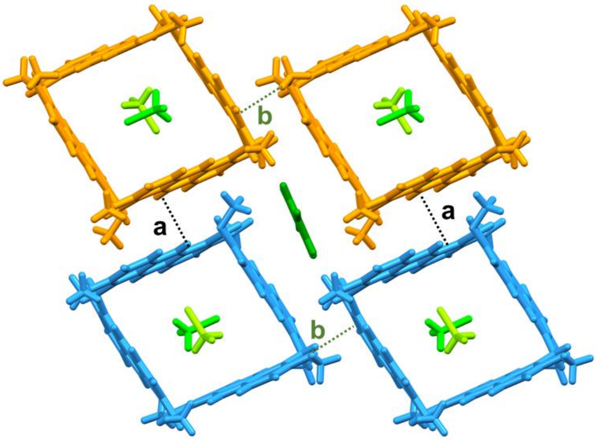

Supplementary Figure 49. Crystal structure of P4-TPN$_b$ showing the different noncovalent interactions between adjacent P4. P4 in color blue represent the crystal structures of *pS*-P4 and the orange color P4 represent *pR*-P4. When the adjacent P4 are enantiomers with each other, perfect parallel [π-π] stacking interactions with distance of 3.51Å (a) and the corresponding dihedral angle of 0° could be observed. For the P4 macrocycles with same configuration, the adjacent P4 interacted with each other by C-H···π interaction with distance of 2.89 Å (b) and angle of 137.44°.

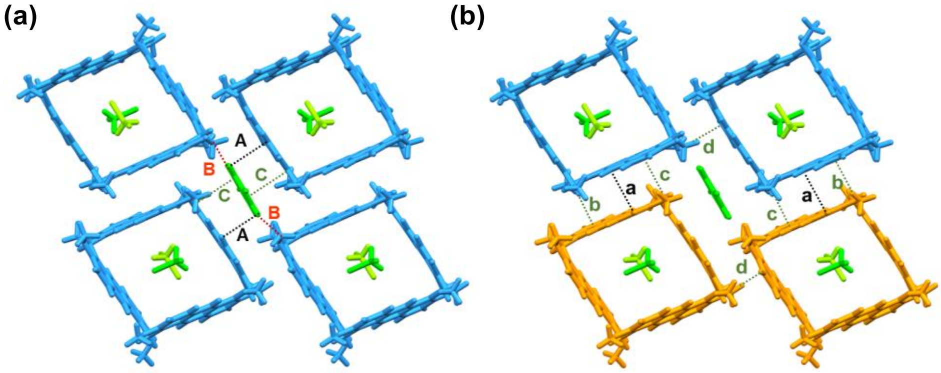

Supplementary Figure 50. Crystal structure of P4-TPN$_b$ showing (a) the parallelogram tiling formed by one TPN-2 and four P4 around through CT and multiple noncovalent interactions. (b) the different noncovalent interactions between adjacent P4. P4 in color blue represent the crystal structures of *pS*-P4 and the orange color P4 represent *pR*-P4. Black, red and green dashed lines indicate π···π interactions (A and a), C-H···O interactions (B) and C-H···π interactions (C, b, c, and d), respectively. Detailed parameters are as follows. The centroid-plane distances (Angstroms): A, 3.39; a, 3.48, and the corresponding dihedral angles (degrees): A, 3.63; a, 0. C-H···O distances

(Angstroms): B, 2.60. C-H···O angles (degrees): B, 172.12. C-H···π distances (Angstroms): C,2.82; b, 2.84; c, 2.84 and d, 2.89. C-H···π angles (degrees): C, 138,74; b, 149.26; c, 149.26 and d, 137.44.

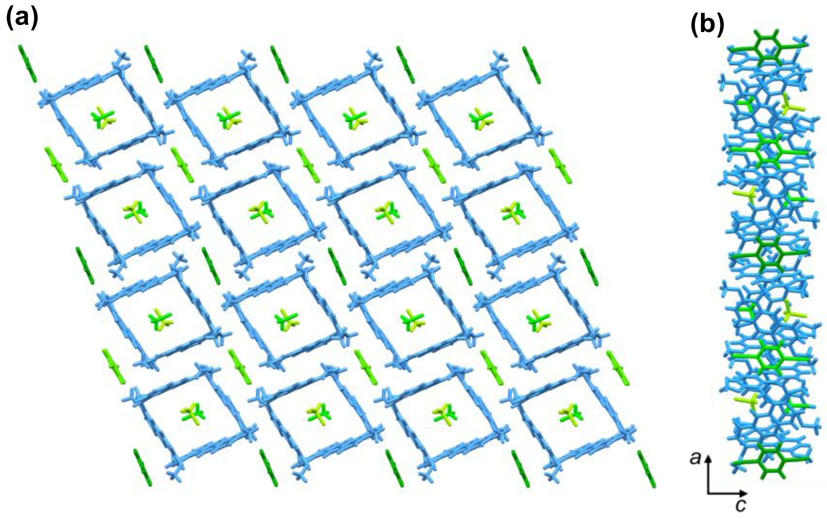

Supplementary Figure 51. Crystal structures of P4-TPN$_b$ showing the layer-like superstructure (a) from top view and (b) in the *ac*-plane. The colors represent the symmetry equivalence and hydrogen atoms are removed for the sake of clarity.

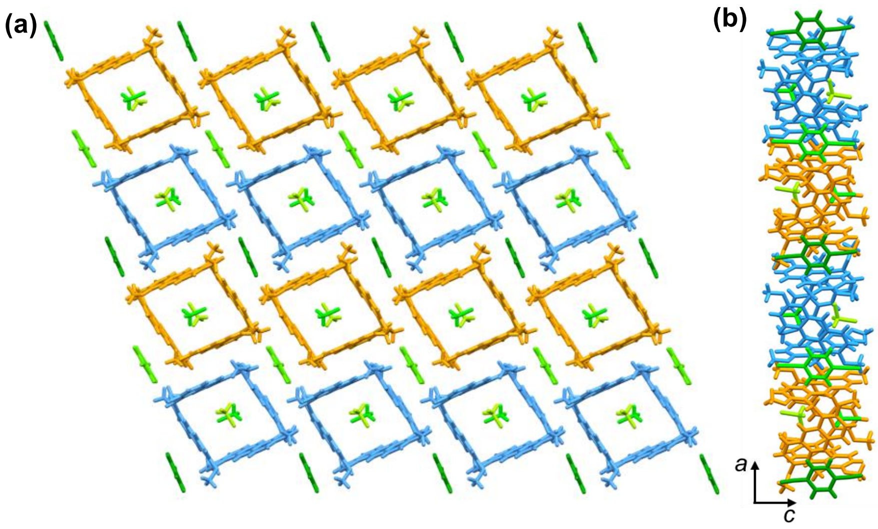

Supplementary Figure 52. Crystal structures of P4-TPN$_b$ showing the layer-like superstructure (a) from top view and (b) in the *ac*-plane. The colors represent the symmetry equivalence except that P4 in color blue represent the crystal structures of *pS*-P4 and the orange color P4 represent *pR*-P4 and hydrogen atoms are removed for the sake of clarity.

5.5 Crystal Structure of P4-TFTN$_a$

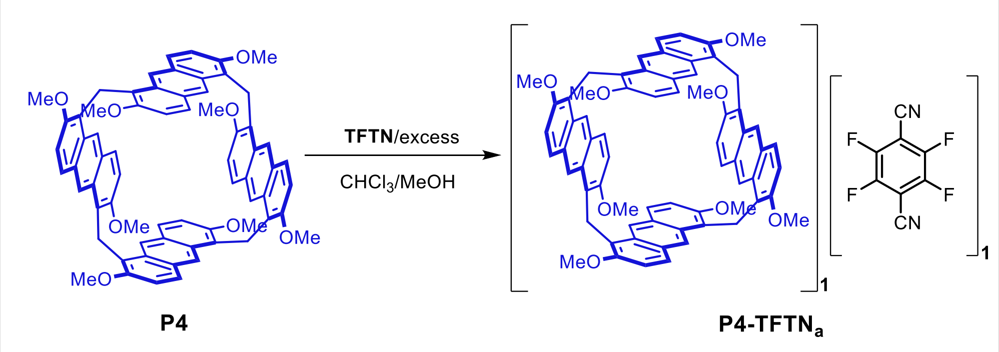

Supplementary Figure 53. Co-crystallization of P4 with TFTN to afford P4-TFTN$_a$.

Method. Red hexgonal crystals were obtained by MeOH vapor diffusion into a 2 mL $CHCl_3$ solution containing P4 (5 mg) and TFTN (5 mg). The crystals were isolated for single crystal X-ray diffraction. The molar ratio of the P4 and TFTN in the crystal structure was 1 : 1.

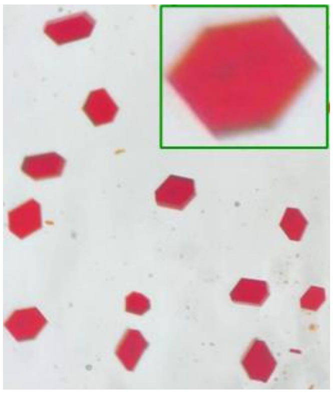

Supplementary Figure 54. Photograph of co-crystal of P4-TFTN$_a$.

Crystal Data. Empirical formula = [($C_{78}H_{58}Cl_6F_4N_2O_8$)], formula weight = 1439.96, crystal system = triclinic, space group = *P*-1, a = 11.2718(2) Å, b = 13.8440(3) Å, c = 23.3425(4) Å, α = 80.372(2) °, β = 84.3120(10) °, γ = 72.391(2) °, V = 3418.38(12) Å^3, Z = 2, T = 169.99(10) K, μ(CuKα) = 2.882 mm^{-1}, D_{calc} = 1.399 g/cm^3, 48102 reflections measured (6.77 ≤ 2Θ ≤ 151.186), 13552 unique (R_{int} = 0.0276, R_{sigma} = 0.0261) which were used in all calculations. The final R_1 was 0.0946 (I > 2σ(I)) and wR_2 was 0.2783 (all data). CCDC number: 2087967.

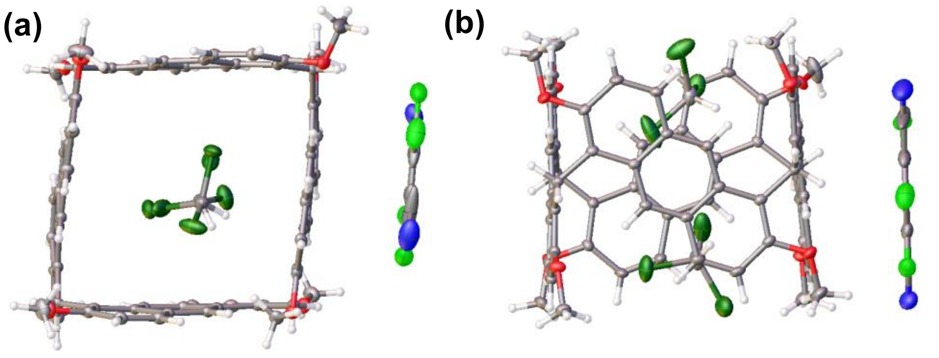

Supplementary Figure 55. ORTEP drawing of P4-TFTN$_a$ from (a) top view and (b) side view (the thermal ellipsoids are displayed at a 30 % probability).

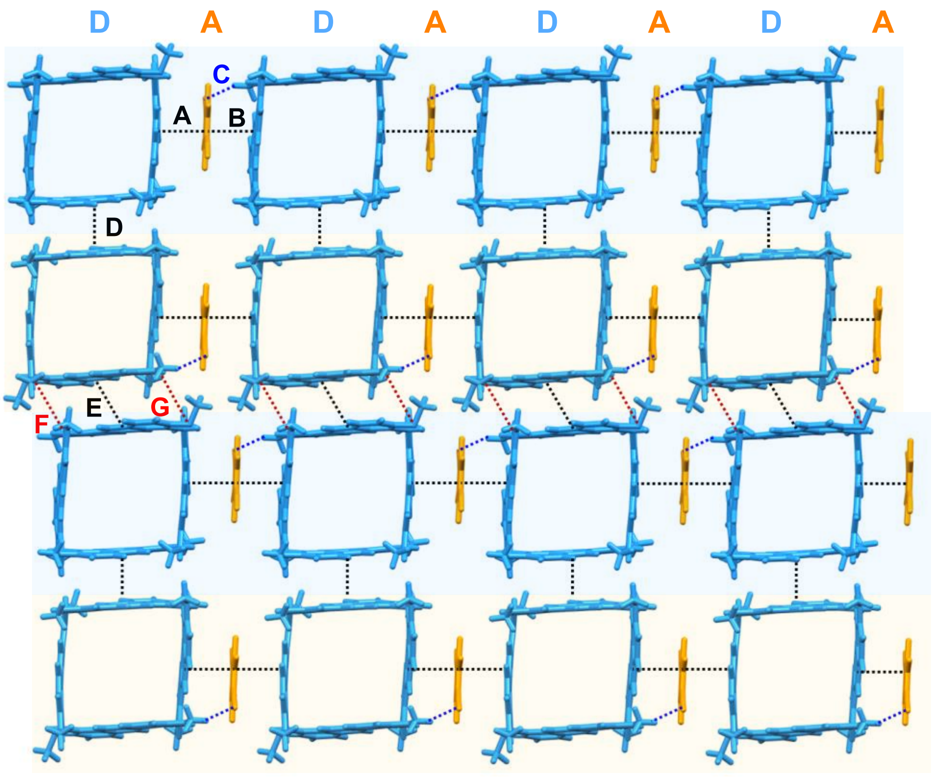

Supplementary Figure 56. 2D layer-like network superstructure of P4-TFTN$_a$. Different colors represent the symmetry equivalence and solvent molecules are removed for the sake of clarity.

5.6 Crystal structure of P4-TFTN$_b$.

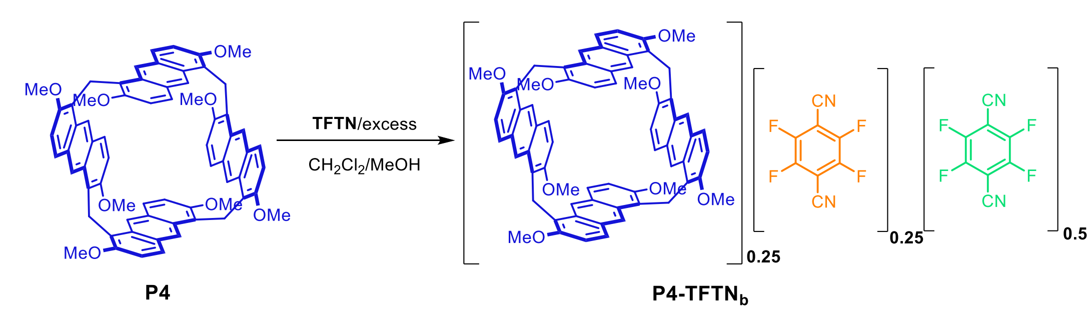

Supplementary Figure 57. Co-crystallization of P4 with TFTN to afford P4-TFTN$_b$.

Method. Red rhombic crystals were obtained by MeOH vapor diffusion into a 2 mL $CHCl_3$ solution containing P4 (5 mg) and TFTN (5 mg). The crystals were isolated for single crystal X-ray diffraction. The molar ratio of the P4 and TFTN in the crystal structure was 1:3.

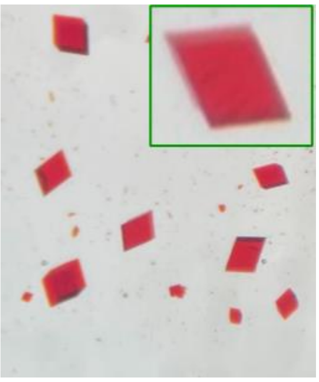

Supplementary Figure 58. Photograph of co-crystal of P4-TFTN$_b$.

Crystal Data. Empirical formula = [($C_{92}H_{54}F_{12}N_6O_8$)], formula weight = 1599.41, crystal system = monoclinic, space group = *C2/m*, a = 22.4953(8) Å, b = 18.4986(5) Å, c = 10.4219(4) Å, α = 90 °, β = 112.381(3) °, γ = 90 °, V = 4010.2(2) Å^3, Z = 2, T = 170.00(13) K, μ(CuKα) = 0.885 mm^{-1}, D_{calc} = 1.325 g/cm^3, 14050 reflections measured ($6.394 \leq 2\Theta \leq 150.752$), 4093 unique ($R_{int}$ = 0.0244, R_{sigma} = 0.0222) which were used in all calculations. The final R_1 was 0.1198 ($I > 2\sigma$(I)) and wR_2 was 0.3393 (all data). CCDC number: 2087969.

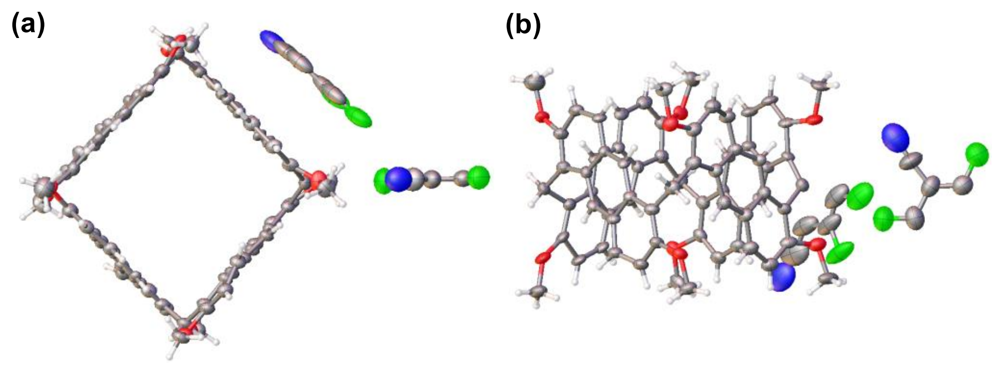

Supplementary Figure 59. ORTEP drawing of P4-TFTN$_b$ from (a) top view and (b) side view (the thermal ellipsoids are displayed at a 30 % probability).

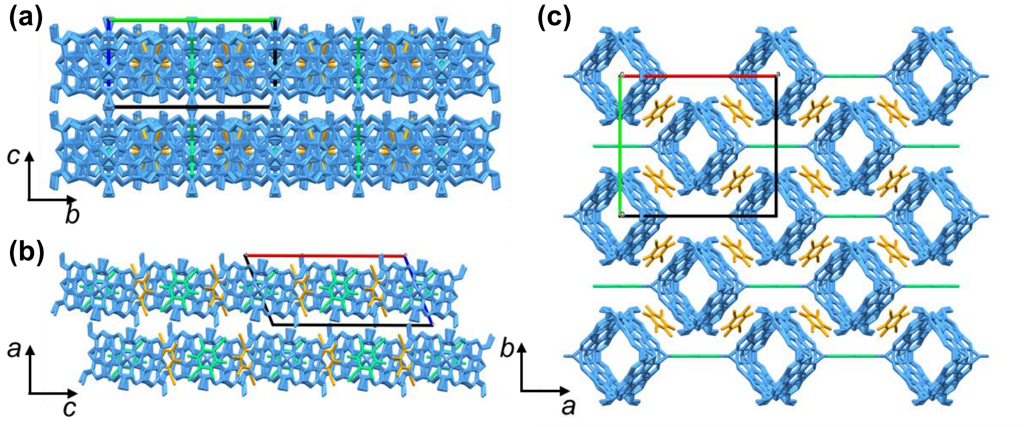

Supplementary Figure 60. Packing mode of P4-TFTN$_b$ viewed along (a) *a*-axis (b) *b*-axis and (c) *c*-axis showing the uniform 2D layer-like rhombic tiling. Different colors represent the symmetry equivalence and hydrogen atoms are omitted for the sake of clarity.

5.7 Crystal Structure of P4-TFTN$_c$

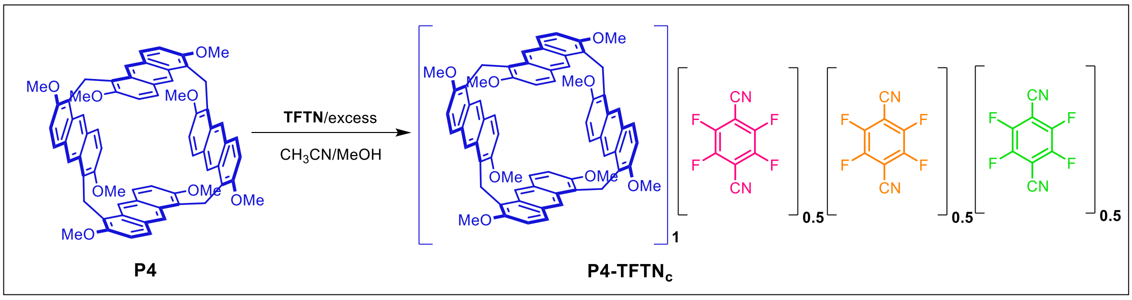

Supplementary Figure 61. Co-crystallization of P4 with TFTN to afford P4-TFTN$_c$.

Method. Red irregular crystals were obtained by MeOH vapor diffusion into a 2 mL CH_3CN solution containing P4 (5 mg) and TFTN (5 mg). The crystals were isolated for single crystal X-ray diffraction. The molar ratio of the P4 and TFTN in the crystal structure was 1 : 1.5.

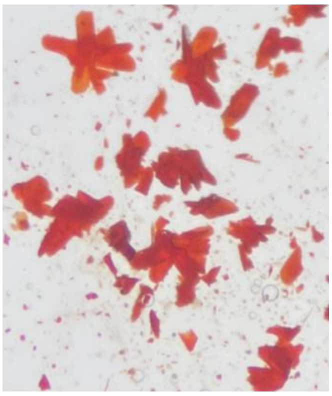

Supplementary Figure 62. Photograph of co-crystal of P4-$TFTN_c$.

Crystal Data. Empirical formula = [($C_{86}H_{65}F_6N_6O_8$)], formula weight = 1424.44, crystal system = triclinic, space group = P-1, a = 13.5789(8) Å, b = 15.1014(12)Å, c = 18.7131(13) Å, α = 100.513(6) °, β = 106.962(6) °, γ = 95.304(6) °, V = 3565.2(5) Å^3, Z = 2, T = 170.00(13) K, μ(CuKα) = 0.798 mm^{-1}, D_{calc} = 1.327 g/cm^3, 53853 reflections measured (5.06 ≤ 2Θ ≤ 151.54), 14133 unique (R_{int} = 0.0668, R_{sigma} = 0.0517) which were used in all calculations. The final R_1 was 0.0931 (I > 2σ(I)) and wR_2 was 0.2667 (all data). CCDC number: 2087970.

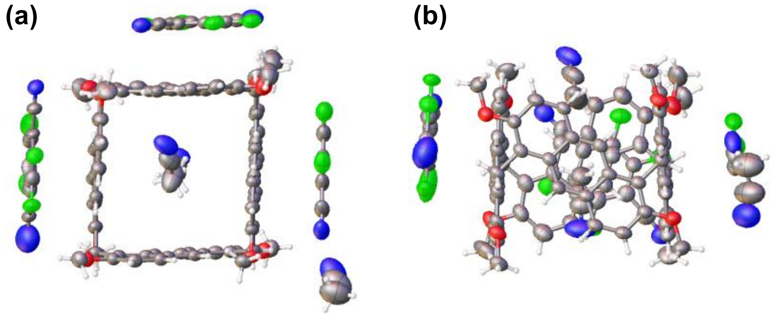

Supplementary Figure 63. ORTEP drawing of P4-$TFTN_b$ from (a) top view and (b) side view (the thermal ellipsoids are displayed at a 30 % probability).

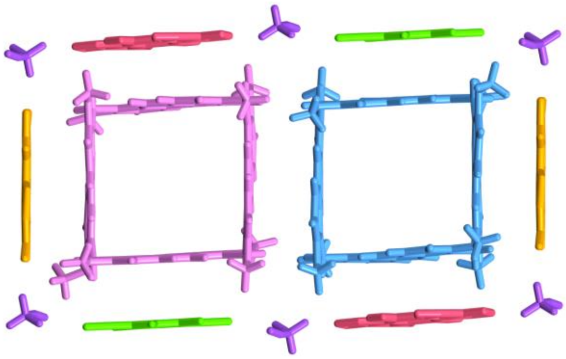

Supplementary Figure 64. The basic tiling unit of P4-TFTN$_c$ formed by two P4, six TFTN molecules around and six CH_3CN molecules as vertexes. Different colors represent the symmetry equivalence except that P4 in color blue represent the crystal structures of *pS*-P4 and the pink color P4 represent *pR*-P4, and solvent molecules are removed for the sake of clarity.

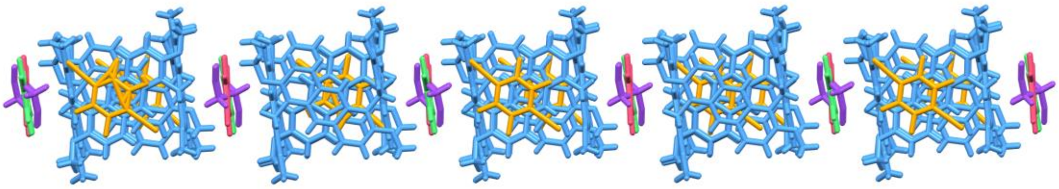

Supplementary Figure 65. Crystal structures of P4-TFTN$_c$ showing the layer-like superstructure. Different colors represent the symmetry equivalence.

Supplementary Table 2. Experimental Crystallographic Data for the Co-crystals of P4-DNB_a and P4-DNB_b.

Compound	P4-DNB_a	P4-DNB_b
Empirical formula	$C_{68}H_{56}O_8{\cdot}C_6H_4N_2O_4{\cdot}CHCl_3$	$C_{68}H_{56}O_8{\cdot}0.5(C_6H_4N_2O_4){\cdot}$ $0.5(C_6H_4N_2O_4){\cdot}2(CH_2Cl_2)$
Formula weight	1288.6	1339.09
Temperature/K	169.99(10)	169.99(11)
Crystal system	monoclinic	triclinic
Space group	$P2_1/n$	P-1
a/Å	23.17880(10)	13.6989(4)
b/Å	22.80780(10)	13.7879(4)
c/Å	25.69640(10)	17.7478(6)
α/°	90	110.554(3)
β/°	104.1010(10)	94.114(3)
γ/°	90	90.114(3)
Volume/Å^3	13175.26(11)	3129.38(18)
Z	8	2
ρ_{calc}g/cm^3	1.299	1.421
μ/mm^{-1}	1.791	2.29
F (000)	5376	1396
Crystal size/mm^3	0.32 × 0.26 × 0.18	0.32 × 0.24 × 0.1
Radiation	CuK_α (λ = 1.54184)	CuK_α (λ = 1.54184)
2Θ range for data collection/°	4.608 to 150.842	5.334 to 151.102
Index ranges	$-29 \le h \le 28$, $-28 \le k \le 28$, $-32 \le l \le 26$	$-16 \le h \le 17$, $-17 \le k \le 17$, $-17 \le l \le 21$
Reflections collected	104377	43262
Independent reflections	26247 [R_{int} = 0.0267, R_{sigma} = 0.0219]	12431 [R_{int} = 0.0413, R_{sigma} = 0.0338]
Data/restraints/parameters	26247/0/1673	12431/0/855
Goodness-of-fit on F^2	1.057	1.485
Final R indexes [I>=2σ (I)]	R_1 = 0.0864, wR_2 = 0.2360	R_1 = 0.1047, wR_2 = 0.3273
Final R indexes [all data]	R_1 = 0.0921, wR_2 = 0.2408	R_1 = 0.1201, wR_2 = 0.3482
Largest diff. peak/hole/eÅ^{-3}	2.38/-2.88	2.65/-1.40

Supplementary Table 3. Experimental Crystallographic Data for the Co-crystals of P4-TPN_a and P4-TPN_b.

Compound	P4-TPN_a	P4-TPN_b
Empirical formula	$C_{68}H_{56}O_8 \cdot C_8H_4N_2 \cdot CHCl_3$	$C_{68}H_{56}O_8 \cdot 0.5(C_8H_4N_2) \cdot 0.5(C_8H_4N_2) \cdot 2(CH_2Cl_2)$
Formula weight	1248.62	1299.11
Temperature/K	169.99(14)	169.99(13)
Crystal system	monoclinic	triclinic
Space group	$P2_1/n$	P-1
a/Å	23.2353(7)	13.6509(4)
b/Å	22.7247(5)	13.8005(4)
c/Å	25.7071(6)	17.8041(4)
α/°	90	69.609(3)
β/°	105.340(3)	86.172(2)
γ/°	90	89.886(2)
Volume/Å^3	13090.1(6)	3136.10(16)
Z	8	2
ρ_{calc}g/cm^3	1.267	1.376
μ/mm^{-1}	1.739	2.219
F (000)	5216	1356
Crystal size/mm^3	0.16 × 0.1 × 0.08	0.27 × 0.24 × 0.16
Radiation	CuK_α (λ = 1.54184)	CuK_α (λ = 1.54184)
2Θ range for data collection/°	4.562 to 132	5.308 to 150.902
Index ranges	-27 ≤ h ≤ 26, -26 ≤ k ≤ 26, -27 ≤ l ≤ 30	-17 ≤ h ≤ 17, -16 ≤ k ≤ 17, -17 ≤ l ≤ 22
Reflections collected	22670	32396
Independent reflections	22670 [R_{int} = 0.0923, R_{sigma} = 0.0641]	12130 [R_{int} = 0.0417, R_{sigma} = 0.0417]
Data/restraints/parameters	22670/151/1675	12130/0/837
Goodness-of-fit on F^2	2.709	1.867
Final R indexes [I>=2σ (I)]	R_1 = 0.1776, wR_2 = 0.4376	R_1 = 0.1114, wR_2 = 0.2699
Final R indexes [all data]	R_1 = 0.2103, wR_2 = 0.4481	R_1 = 0.1412, wR_2 = 0.2899
Largest diff. peak/hole/eÅ^{-3}	0.78/-0.94	1.01/-0.89

Supplementary Table 4. Experimental Crystallographic Data for the Co-crystals of P4-TFTN$_a$, P4-TFTN$_b$ and P4-TFTN$_c$.

Compound	P4-TFTN$_a$	P4-TFTN$_b$	P4-TFTN$_c$
Empirical formula	$C_{68}H_{56}O_8 \cdot C_8F_4N_2 \cdot 2(CHCl_3)$	$C_{68}H_{56}O_8 \cdot (C_8F_4N_2) \cdot 2(C_8F_4N_2)$	$C_{68}H_{56}O_8 \cdot 0.5(C_8F_4N_2) \cdot 0.5(C_8F_4N_2) \cdot 0.5(C_8F_4N_2) \cdot 3(C_2H_3N)$
Formula weight	1439.96	1599.41	1424.44
Temperature/K	169.99(10)	170.00(2)	170.00(13)
Crystal system	triclinic	monoclinic	triclinic
Space group	*P*-1	*C*2/*m*	*P*-1
a/Å	11.2718(2)	22.4953(8)	13.5789(8)
b/Å	13.8440(3)	18.4986(5)	15.1014(12)
c/Å	23.3425(4)	10.4219(4)	18.7131(13)
α/°	80.372(2)	90	100.513(6)
β/°	84.3120(10)	112.381(3)	106.962(6)
γ/°	72.391(2)	90	95.304(6)
Volume/Å^3	3418.38(12)	4010.2(2)	3565.2(5)
Z	2	2	2
ρ_{calc}g/cm^3	1.399	1.325	1.327
μ/mm^{-1}	2.882	0.885	0.798
F (000)	1484	1640	1482
Crystal size/mm^3	0.24 × 0.22 × 0.2	0.12 × 0.1 × 0.08	0.12 × 0.08 × 0.03
Radiation	CuK$_\alpha$ (λ = 1.54184)	CuK$_\alpha$ (λ = 1.54184)	CuK$_\alpha$ (λ = 1.54184)
2Θ range for data collection/°	6.77 to 151.186	6.394 to 150.752	5.06 to 151.54
Index ranges	$-14 \le h \le 14, -17 \le k \le 16, -29 \le l \le 26$	$-27 \le h \le 27, -14 \le k \le 22, -13 \le l \le 12$	$-16 \le h \le 16, -18 \le k \le 18, -23 \le l \le 22$
Reflections collected	48102	14050	53853
Independent reflections	13552 [R_{int} = 0.0276, R_{sigma} = 0.0261]	4093 [R_{int} = 0.0244, R_{sigma} = 0.0222]	14133 [R_{int} = 0.0668, R_{sigma} = 0.0517]
Data/restraints/ parameters	13552/0/891	4093/649/785	14133/412/1102

Goodness-of-fit on F^2	1.048	2.459	1.289
Final R indexes [I>=2σ (I)]	R_1 = 0.0946, wR_2 = 0.2726	R_1 = 0.1198, wR_2 = 0.3243	R_1 = 0.0931, wR_2 = 0.2270
Final R indexes [all data]	R_1 = 0.1001, wR_2 = 0.2783	R_1 = 0.1465, wR_2 = 0.3393	R_1 = 0.1799, wR_2 = 0.2667
Largest diff. peak/hole/eÅ^{-3}	1.90/-1.32	1.19/-0.48	0.91/-0.45
